# O‐GalNAc Glycosylation Activates MBL‐Mediated Complement and Coagulation Cascades to Drive Organotropic Metastasis

**DOI:** 10.1002/advs.202504809

**Published:** 2025-06-10

**Authors:** Xinyu Chen, Wei Bao, Kaiyuan Liu, Na Jing, Genyu Du, Luyao Jiang, Qian You, Yingchao Zhang, Penghui Xu, Chaping Cheng, Nan Wang, Xialian Xi, Mingyue Wang, Yiyun Liu, Jinming Wang, Huifang Zhao, Shilei Zhang, Dinglan Wu, Chi‐Fai Ng, Jiahua Pan, Wei Xue, Wei‐Qiang Gao, Pengcheng Zhang, Kai Zhang, Helen He Zhu

**Affiliations:** ^1^ State Key Laboratory of Systems Medicine for Cancer Department of Urology Ren Ji Hospital Shanghai Cancer Institute Shanghai Jiao Tong University School of Medicine Shanghai 200127 China; ^2^ Med‐X research Institute School of Biomedical Engineering Shanghai Jiao Tong University Shanghai 200030 China; ^3^ Department of Pathology Ren Ji Hospital Shanghai Jiao Tong University School of Medicine Shanghai 200127 China; ^4^ Department of Surgery Faculty of Medicine The Chinese University of Hong Kong Hong Kong 999077 China; ^5^ S.H. Ho Urology Centre Department of Surgery Prince of Wales Hospital The Chinese University of Hong Kong Hong Kong 999077 China; ^6^ Department of Urology Renji Hospital Shanghai Jiao Tong University School of Medicine Shanghai 200127 China; ^7^ School of Biomedical Engineering Shanghai Tech University Shanghai 201210 China

**Keywords:** liver metastasis, O‐GalNAc glycosylation, glant9, neuroendocrine prostate cancer, mannose binding lectin (MBL), complement

## Abstract

Liver metastasis is prevalent among patients with neuroendocrine prostate cancer (NEPC) and other types of neuroendocrine (NE) cancers, featuring with an aggressive clinical course and a dismal prognosis. However, the cellular and molecular mechanisms underlying liver‐specific metastatic tropism in NE cancers remain poorly understood. Intriguingly, it is found that NEPC liver metastatic foci are frequently associated with thrombi. NEPC cells express an aberrantly elevated level of glycosyltransferase Galnt9. Notably, the Galnt9‐mediated O‐GalNAc glycosylation on the cell membrane of NE cancer cells, particularly on the adhesion molecule Annexin A2, activates the mannose‐binding lectin (MBL) complement signaling in the liver. This cascade stimulates platelet activation and thrombus formation, ultimately facilitating hepatic metastasis of NEPC. Inhibition of O‐GalNAc glycosylation or knockdown of *Galnt9* demonstrates efficacy in restraining the liver metastasis of NEPC, small cell lung cancer (SCLC), and colorectal neuroendocrine cancer. These findings identify Galnt9‐mediated O‐GalNAc glycosylation as a targetable mechanism driving liver metastasis through activation of MBL complement and coagulation cascades across a broad spectrum of NE cancers.

## Graphical abstract

Upregulated glycosyltransferase GALNT9 in neuroendocrine (NE) carcinomas increases O‐GalNAc glycosylation on cell membrane proteins, particularly Annexin‐A2 (ANXA2). Elevated O‐GalNAc glycan induces the binding of NE cancer cells to mannose binding lectin 2 (MBL2), activating the MBL‐MBL associated serine protease (MBL‐MASP) complement pathway in the liver, triggering platelet activation and thrombus formation, ultimately facilitating liver metastasis in neuroendocrine prostate cancer (NEPC).

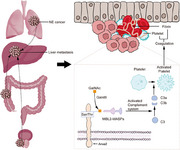

## 1 Introduction

Organotropic metastasis is orchestrated by the interplay between the target organ and the tumor cells. The liver, with abundant blood supply from the hepatic artery and the portal vein, provides an optimal environment for the colonization and growth of tumor cells. Clinical observations have shown that neuroendocrine prostate cancers (NEPC) exhibit a marked tendency for liver metastasis, whereas prostate adenocarcinoma (PrAD) preferentially metastasizes to bone.^[^ ^1^ ^]^ Of note, neuroendocrine (NE) cancers originating from other primary sites, such as the lung and colon, also demonstrate a liver‐specific metastatic tropism.^[^ ^2^ ^]^ NE cancer liver metastases are associated with a much shorter patient survival compared to metastases in other locations.^[^ ^3^ ^]^ Thus, identifying targetable molecular mechanisms of liver metastasis in NE cancers is crucial for developing therapies to improve clinical outcomes.

Clinical studies show that patients with NE cancers have an increased risk of developing thrombosis.^[^ ^4^ ^]^ Platelet activation, a key step in thrombosis, has been implicated in facilitating cancer metastasis.^[^ ^5^ ^]^ For instance, platelets are found to protect tumor cells from shear forces and attacks by natural killer (NK) cells.^[^ ^6^ ^]^ In addition, platelets or platelet‐derived microparticles enhance cancer cells adhesion to the vascular endothelium.^[^ ^7^ ^]^ Despite these findings, there is still a lack of studies on how upstream mechanisms stimulate platelet activation and precipitate thrombosis during organ‐specific metastasis, especially in liver metastasis of NE cancers.

Thrombosis and complement activation are often closely linked and mutually reinforcing.^[^ ^8^ ^]^ The complement activation process is characterized by a series of cascade reactions catalyzed by serine proteases. There are three principal pathways for complement activation: the classical pathway, the alternative pathway, and the mannose‐binding lectin (MBL) pathway.^[^ ^9^ ^]^ The classical pathway is primarily initiated by antigen‐antibody complexes, occurring predominantly in the late stage of infection.^[^ ^10^ ^]^ The alternative pathway is primarily activated by the bacterial cell wall component lipopolysaccharide.^[^ ^11^ ^]^ The MBL pathway is typically initiated by glycosylated surface components of exogenous pathogens.^[^ ^12^ ^]^ Glycosylation changes have been shown to occur during tumorigenesis.^[^ ^13^ ^]^ Whether the altered glycosylation status of cancer cells can also induce the MBL complement cascade remains unknown. Traditionally, high‐mannose glycans have been recognized as the primary structures that activate MBL.^[^ ^14^ ^]^ However, the potential for other glycans to activate MBL remains largely unexplored, particularly in pathological states where mannose glycan is not the predominant form. In this study, we investigate whether neuroendocrine cancer cells possess distinctive attributes that facilitate complement activation and explore the role of the complement pathway in thrombosis and liver metastasis of NE cancers, with a particular focus on NEPC.

Here, we find that the glycosyltransferase GALNT9 is highly expressed in NEPC, small cell lung cancer (SCLC), and neuroendocrine colon cancers, leading to an increased presence of O‐GalNAc glycosylation on the cell surface. The highly glycosylated cell membrane activates the MBL complement pathway and initiates platelet activation and thrombus formation, facilitating liver metastasis in NE carcinomas.

## 2 Results

### 2.1 Small Thrombi are Often Found in Close Proximity to Liver Metastatic Foci at the Early Metastatic Stage in NEPC Tumor‐Bearing Mice

**Figure 1:**
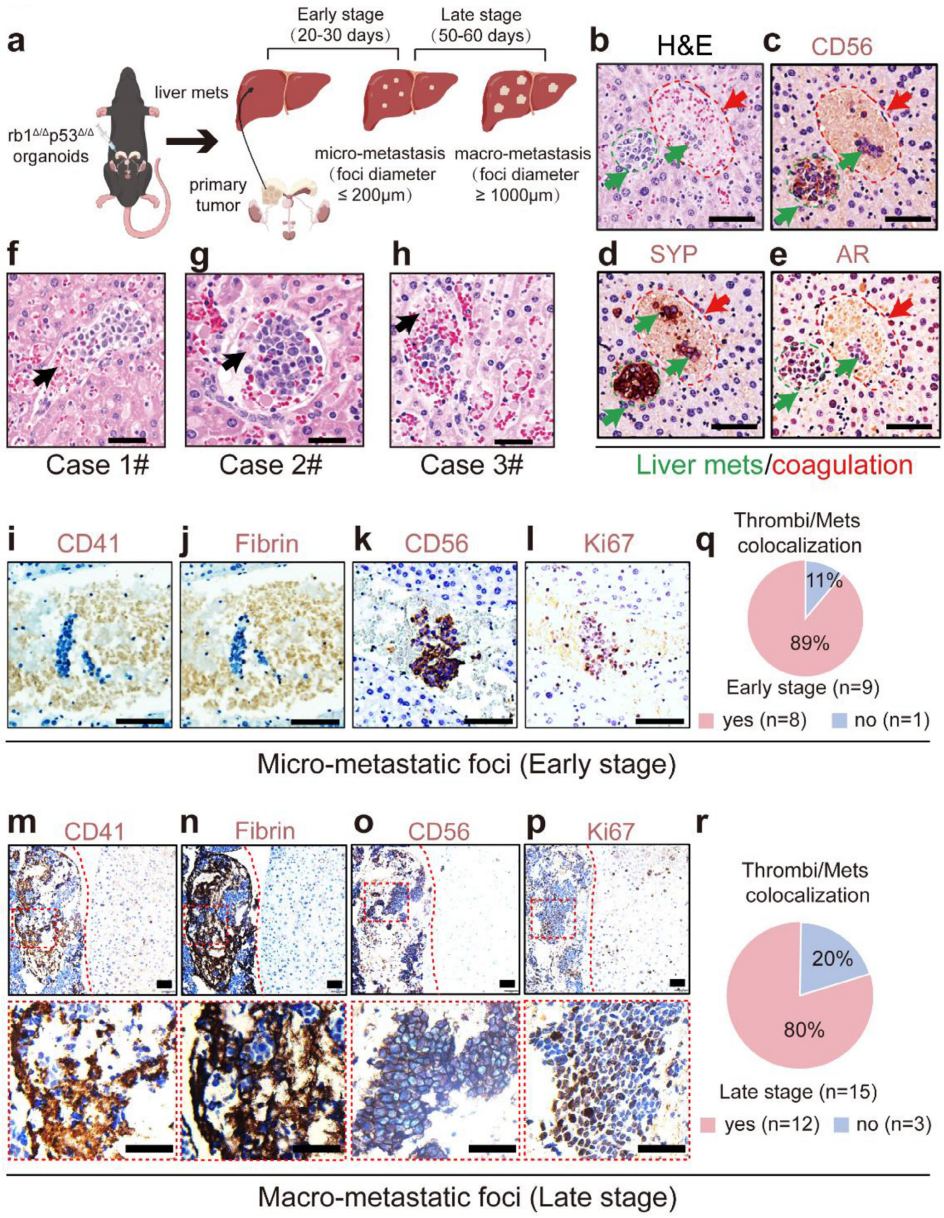
Thrombi are frequently in close proximity to liver metastatic foci in NEPC tumor‐bearing mice. a) The schematic diagram of the animal model for liver metastasis of NEPC via orthotopic inoculation of *rb1* ^Δ/Δ^ *p53* ^Δ/Δ^ murine NEPC organoids into the prostate of WT C57BL/6 mice. b–e) H&E and IHC c–e) staining showing that the CD56^+^SYP^+^AR^‐^ micro‐metastatic foci are attached to or surrounded by thrombi in the liver of NEPC‐tumor bearing mice at the early metastatic stage. Scale bar = 100 µm. f–h) H&E staining showing that the micro‐metastatic foci are attached to or surrounded by thrombi in the liver of 3 individual NEPC‐tumor bearing mice at the early metastatic stage. Scale bar = 50 µm i–l) IHC staining against CD41 and Fibrin confirms thrombi aggregations around micro‐metastatic NEPC tumor proliferating cell clusters at the early metastatic stage. Scale bar = 50 µm m,n) IHC staining against CD41 and Fibrin confirms thrombi aggregations adjacent to macro‐metastatic tumor cell clusters at the late metastatic stage (scale bar = 100 µm, upper panel). Indicative areas are enlarged to represent a detailed colocalization between thrombi and liver metastasis. (scale bar = 100 µm, lower panel). o,p) IHC staining against the neuroendocrine markers CD56 and the proliferating marker Ki67, validating the identity of NEPC cells at the late metastatic stage (scale bar = 100 µm).q,r) Quantification results showing that 8 out of 9 *rb1* ^Δ/Δ^ *p53* ^Δ/Δ^ NEPC tumor‐bearing mice at the early metastatic stage and 12 out of 15 *rb1* ^Δ/Δ^ *p53* ^Δ/Δ^ NEPC tumor‐bearing mice at the late metastatic stage exhibit evident colocalization between thrombi and liver metastasis.

Previously, we established a stable NEPC liver metastasis model via orthotopic inoculation of prostate cancer (PCa) organoids established from *PbsnCre^+^; Rb1^f/f^; Trp53^f/f^* (*rb1^Δ/Δ^p53^Δ/Δ^*) NEPC model mice into the prostates of wild‐type (WT) C57BL/6 mice^[^ ^15^ ^]^ (**Figure** **1**a). In this model, hepatic micrometastasis with an average foci diameter of less than 200 µm was readily detected at 20–30 days after orthotopic inoculation. We designated this period as the early metastatic stage (Figure 1a). As the disease progressed, macrometastases in the liver with average foci diameters exceeding 1000 µm were observed at 50–60 days post‐implantation, and this period was designated the late metastatic stage (Figure 1a). Using serial paraffin‐embedded liver sections from NEPC tumor‐bearing mice, hematoxylin & eosin (H&E, Figure 1b) and immunohistochemical (IHC) staining revealed neural cell adhesion molecule‐1 (CD56, Figure 1c) and synaptophysin (SYP, Figure 1d)‐positive but androgen receptor (AR, Figure 1e)‐negative (CD56^+^SYP^+^AR^‐^) NEPC liver metastatic foci, validating the high penetrance of prostate‐to‐liver metastasis (Figure 1b–e).

Notably, many small thrombi, characterized by the aggregation of fibrin, platelets, and red blood cells, were observed to be attached or close to micrometastatic foci (Figure 1f–h). To comprehensively depict this phenomenon, we also conducted IHC staining for CD41, a platelet marker, and fibrin and observed that small clusters of CD56^+^ proliferating (Ki67^+^) NEPC cells were frequently attached to or surrounded by CD41‐ and fibrin‐expressing thrombi at the early metastatic stage (Figure 1i–l). We further asked whether this phenomenon can be detected in macrometastatic lesions at the late metastatic stage. Consistently, we performed IHC staining for platelet markers and found that macrometastatic foci were also often adhered to or encased by CD41^+^ (Figure 1m) and fibrin^+^ (Figure 1n) thrombi at the late metastatic stage (Figure 1o,p). Quantification revealed that 8 out of 9 mice examined at the early stage (Figure 1q) or 12 out of 15 mice at the late stage (Figure 1r) displayed evident colocalization between thrombi and metastatic foci in the liver. Therefore, these data suggest that thrombus formation may play a role in NEPC liver metastasis.

### 2.2 Complement and Coagulation Cascades are Molecular Hallmarks of NEPC Liver Metastasis

**Figure 2:**
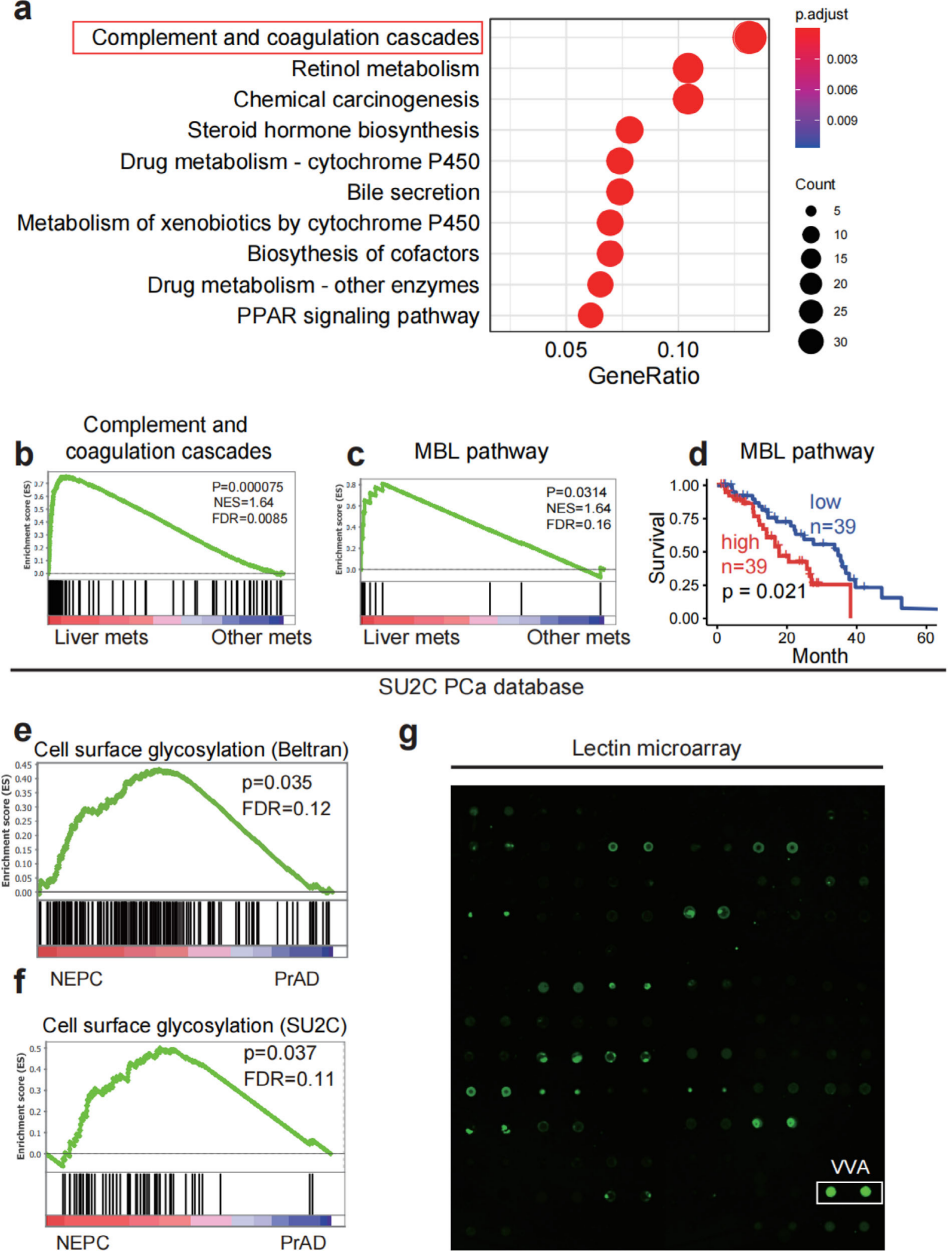
High MBL pathway activity is enriched in PCa liver metastasis and GalNAc is a major glycosylation form on NEPC cell surface. a) The KEGG analysis showing that the “complement and coagulation cascades” is most upregulated signature in liver metastasis versus primary prostate tumors of *rb1* ^Δ/Δ^ *p53* ^Δ/Δ^ NEPC tumor‐bearing mice (n = 3, mice). b,c) GSEA plots reveal that “complement and coagulation cascade” (b) and “MBL pathway activity” are significantly elevated in liver metastatic lesions compared to metastasis in other anatomic sites based on analysis of the SU2C prostate cancer dataset. d) Kaplan–Meier survival analysis of the SU2C prostate cancer dataset reveal that PCa patients with high MBL activity (n = 39) exhibit significantly shorter survival compared to PCa patients with low MBL activity (n = 39). The log rank‐test was applied for statistics. e,f) GSEA plots showing that the “cell surface glycosylation” is significantly elevated in NEPC compared to PrAD based on analysis of Beltran and SU2C prostate cancer datasets. g) Lectin microarray results that the O‐GalNAc glycosylation, as revealed by the strongest signal of vicia villosa (VVA) lectin toward the membrane fraction extracted from murine *rb1* ^Δ/Δ^ *p53* ^Δ/Δ^ NEPC organoids.

To dissect the association between thrombosis and liver metastasis, we carefully isolated primary tumors derived from *rb1* ^Δ/Δ^ *p53* ^Δ/Δ^ organoids and their derivative liver metastases to conduct bulk RNA sequencing (RNA‐seq) experiments. Using Kyoto Encyclopedia of Genes and Genomes (KEGG) and Gene Ontology (GO) analyses, we found that “complement and coagulation cascades” was the most upregulated molecular signature of liver metastases in comparison with primary tumors (**Figure** **2**a). Using gene set enrichment analysis (GSEA), we found that coagulation‐related molecular signatures, such as “blood coagulation fibrin clot formation”, “positive regulation of coagulation” and “hallmark of coagulation”, were also significantly upregulated in the liver metastases of *rb1* ^Δ/Δ^ *p53* ^Δ/Δ^ NEPC tumor‐bearing mice compared with their primary tumors (Figure S1a–c, Supporting Information). Next, we used the Stand Up to Cancer (SU2C) human PCa dataset to perform KEGG and GO analyses. As shown in Figure 2b, human PCa liver metastatic lesions also featured the “complement and coagulation cascades” signature, in contrast to metastases at other anatomic sites. Moreover, “blood coagulation fibrin clot formation”, “positive regulation of coagulation” and “hallmark of coagulation” were prominently present in liver metastases versus primary tumors in the Beltran and SU2C clinical PCa datasets (Figure S1d–i, Supporting Information). These data suggest a species‐conserved molecular signature of NEPC liver metastasis from mice to humans.

Complement activation and coagulation play pivotal roles in thrombosis, which has been demonstrated to be positively implicated in cancer metastasis.^[^ ^16^ ^]^ We, therefore, investigated how complement activation and the coagulation cascade are preferentially activated in NEPC and their functions in promoting NEPC liver metastasis. Using the SU2C PCa dataset,^[^ ^17^ ^]^ we conducted GSEA. Among the three pathways that activate complement cascade signaling, the MBL signature genes (Figure 2c), but not classic (Figure S2a, Supporting Information) or alternative (Figure S2b, Supporting Information) pathway‐related genes, were significantly upregulated in liver metastases compared with other metastases. In addition to the SU2C PCa dataset, we obtained similar results from another clinical PCa dataset, the Beltran PCa RNA‐seq dataset,^[^ ^18^ ^]^ as exemplified by activated complement and coagulation cascades in liver metastatic foci (Figure S2c, Supporting Information). Similarly, only the MBL pathway hallmark genes were significantly enriched in liver metastases compared with other metastatic lesions (Figure S2d–f, Supporting Information).

Next, we explored the correlations of these three pathways with the survival of patients with PCa. Intriguingly, we found that patients with high MBL signal activity presented significantly shorter overall survival (Figure 2d). In contrast, neither the classical pathway (Figure S2g, Supporting Information) nor the alternative pathway (Figure S2h, Supporting Information) correlated with patient survival. Given the clinical relevance of the MBL pathway, MBL‐mediated complement activation is hypothesized to be positively involved in NEPC liver metastasis.

### 2.3 NEPC Cells Bind to MBL and Activate the Complement Cascade

To investigate the hypothesis that NEPC tumor cells are also capable of activating the MBL complement pathway through the glycosylated cell membrane and to understand the difference in the intrinsic attributes between NEPC and prostate adenocarcinoma (PrAD), which may lead to distinct organotropic metastasis of these two PCa subtypes, we first analyzed the Beltran and SU2C PCa transcriptome datasets.^[^ ^18^ ^]^ Compared with their PrAD counterparts, the human NEPC biospecimens in both datasets presented a significant hallmark of “cell surface glycosylation” (Figure 2e,f). To further determine the glycosylation on the NEPC cell surface, we performed a lectin microarray assay to detect the specific lectin binding activity toward carbohydrate residues. Among a total of 70 lectins, including mannose, galactose/*N*‐acetylgalactosamine, *N*‐acetylglucosamine, fucose, and sialic acid, the vicia villosa (VVA) lectin, which specifically binds to GalNAc glycosylation, demonstrated the strongest affinity toward *rb1* ^Δ/Δ^ *p53* ^Δ/Δ^ NEPC cancer cells (Figure 2g), suggesting that the predominant glycosylation on the NEPC cell surface is GalNAc glycosylation.

**Figure 3:**
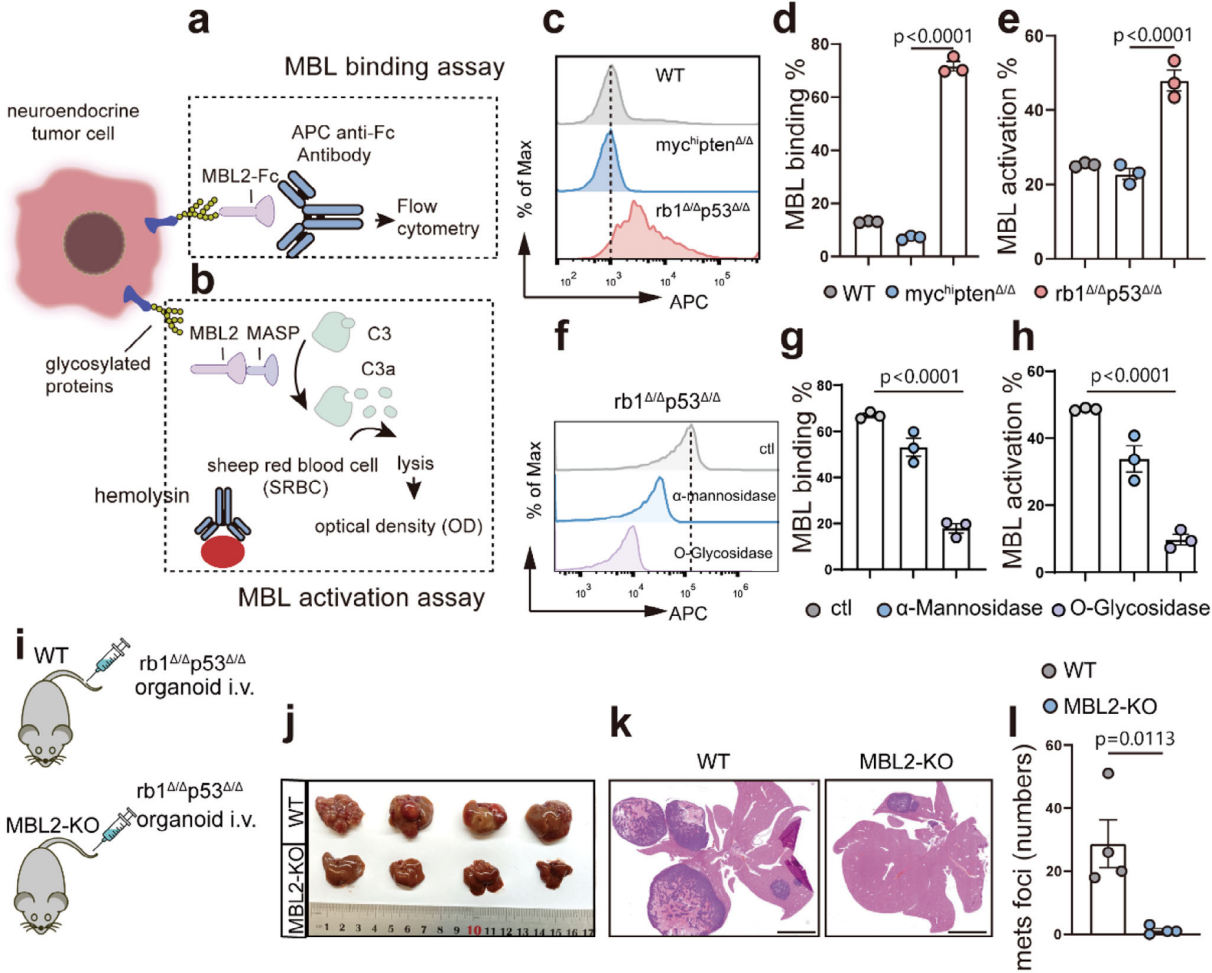
NEPC cells bind to MBL and activate the complement cascade. a,b) Schematic diagrams of experimental design for MBL binding and MBL activation assays. c,d) Flow cytometric analysis and quantification results showing the MBL binding capability of WT prostate epithelial organoids, *myc* ^hi^ *pten* ^Δ/Δ^ PrAD, and *rb1* ^Δ/Δ^ *p53* ^Δ/Δ^ NEPC tumor organoids. e) Quantification data showing the MBL activation capability of WT prostate epithelial organoids, *myc* ^hi^ *pten* ^Δ/Δ^ PrAD, and *rb1* ^Δ/Δ^ *p53* ^Δ/Δ^ NEPC tumor organoids. f–h) Flow cytometric analysis and quantification results showing that the effect of O‐glycosidase and/or α‐mannosidase treatment on MBL binding (f‐g) and activation activities (h) of *rb1* ^Δ/Δ^ *p53* ^Δ/Δ^ NEPC organoids. i) A schematic strategy reveals that the *rb1* ^Δ/Δ^ *p53* ^Δ/Δ^ NEPC organoids were intravenously (i.v.) injected into WT and MBL2‐knockout (MBL2‐KO) mice, validating the essential role of MBL in mediating liver metastasis in NEPC tumor‐bearing mice. j‐l) Dissected livers, H&E staining, and quantification of the liver metastatic foci number of WT and MBL2‐KO mice intravenously inoculated with *rb1* ^Δ/Δ^ *p53* ^Δ/Δ^ NEPC organoids (*n* = 4, mice). For statistics in this figure, the two‐tail unpaired Student's‐*t* test was applied for, and the one‐way ANOVA test was applied for (d‐e) and (g‐h). Data were shown as means ± SD.

We next sought to determine whether NEPC cells possess a greater capacity than PrAD cells to trigger MBL signaling activation via MBL binding (**Figure** **3**a) and activation (Figure 3b) assays. In the MBL binding assay (Figure 3a), cancer cells were incubated with MBL‐Fc, followed by an anti‐Fc‐allophycocyanin (APC) antibody. MBL binding was measured by flow cytometry, which was used to quantify the proportion of APC‐positive cells. To assess the capacity of NEPC cells to activate the MBL–MASP complement pathway, a hemolysis assay was conducted using sheep red blood cells (sRBCs). Cancer cells were incubated with MBL, MASPs, and complement 3 (C3) protein to trigger complement activation (Figure 3b). Hemolysis of sRBCs, induced by activated complement components, was used to measure the extent of activation. As shown in Figure 3c,d, the *rb1* ^Δ/Δ^ *p53* ^Δ/Δ^ NEPC organoids presented significantly stronger MBL binding than did the WT prostate organoids and *myc* ^hi^ *pten* ^Δ/Δ^ PrAD organoids. In line with these data, *rb1* ^Δ/Δ^ *p53* ^Δ/Δ^ NEPC organoids also exhibited a greater capacity to trigger MBL–MASP complement activation than their *myc* ^hi^ *pten* ^Δ/Δ^ PrAD counterparts did (Figure 3e).

Next, to elucidate which form of glycosylation in NEPC results in MBL binding and activation, we conducted KEGG and GO analyses on the SU2C and Beltran human PCa datasets. Among the various forms of glycosylation, O‐GalNAc and O‐mannose were the top two glycosylation types on NEPC compared with PrAD in both datasets (Figure S3a,b, Supporting Information). We then used mannosidase and glycosidase to block O‐GalNAc and O‐mannose glycosylation, respectively. The suppression of MBL binding and activation was much more pronounced following glycosidase treatment than following mannosidase treatment (Figure 3f–h). These data are in line with previous lectin microarray results (Figure 2g) and suggest that O‐GalNAc in NEPC cells is a major contributor to MBL activation.

We then asked whether the MBL pathway is required for liver metastasis of NEPC. To do this, we utilized MBL2 knockout (MBL2‐KO) mice, in which the *rb1* ^Δ/Δ^ *p53* ^Δ/Δ^ organoids were intravenously (i.v.) inoculated to induce liver metastasis (Figure 3i). Compared with WT mice, MBL2‐KO recipients presented a significantly attenuated liver metastatic burden (Figure 3j–l), as evidenced by significant reductions in the number of metastatic foci (Figure 3l). In summary, our data, including those of in vitro MBL binding and activation assays and in vivo NEPC intravenous inoculation experiments, collectively demonstrated that O‐GalNAc‐mediated MBL activation plays an essential role in driving NEPC liver metastasis.

### 2.4 The Upregulated Glycosyltransferase GALNT9 is Essential for NEPC to Activate the MBL Pathway

**Figure 4:**
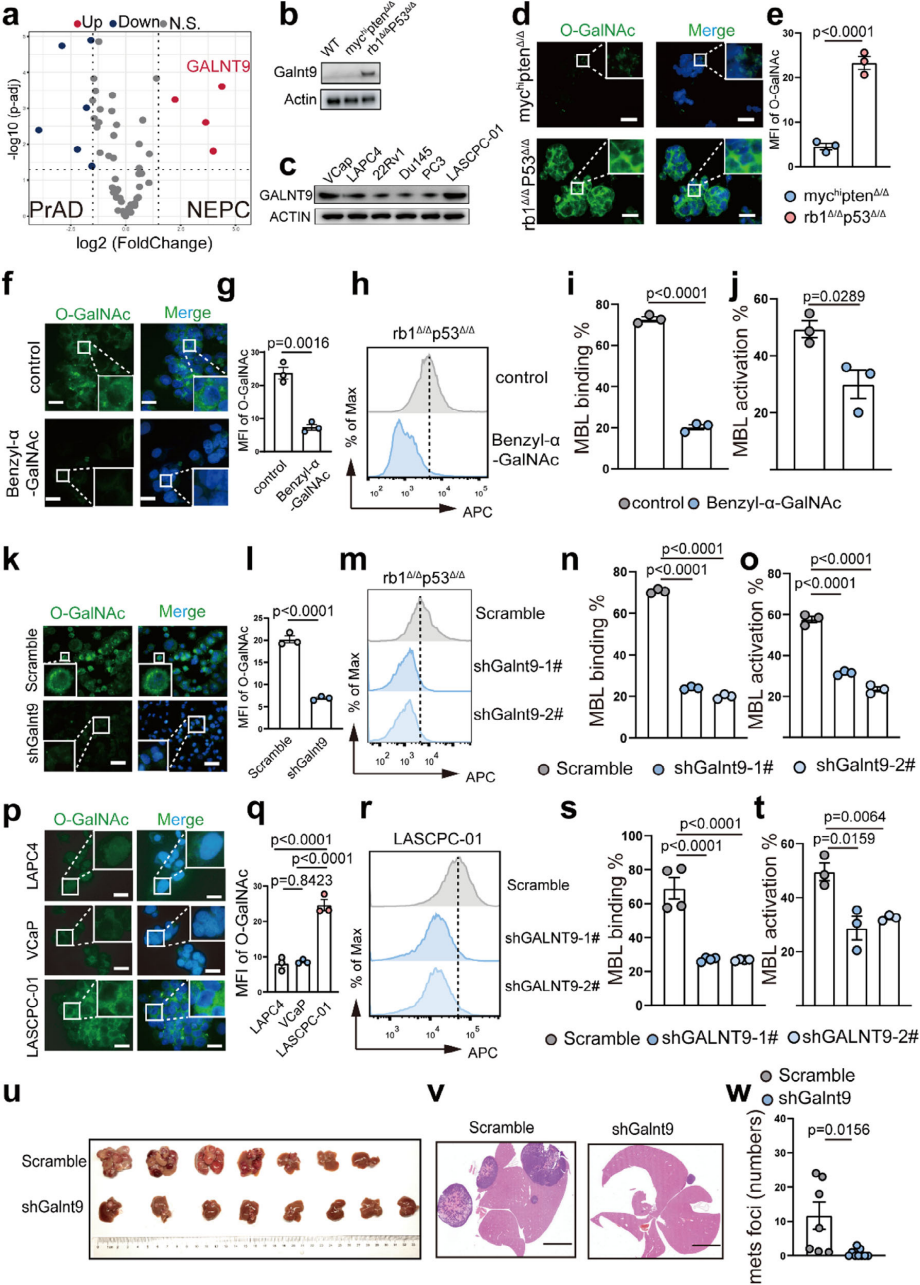
Inhibition of O‐GalNAc glycosylation or Galnt9‐KD attenuates MBL binding and activation and inhibits liver metastasis in NEPC. a) The volcano pot depicts the upregulated, downregulated, and unchanged genes, which encode the enzymes involved in the first two steps of glycosylation based on Beltran human prostate cancer dataset. b) Immunoblots showing the protein level of Galnt9 in WT prostate, *myc* ^hi^ *pten* ^Δ/Δ^ PrAD, and *rb1* ^Δ/Δ^ *p53* ^Δ/Δ^ NEPC organoids. c) Immunoblots showing the protein level of GALNT9 in VCaP, LAPC4, 22Rv1, Du145, PC3 and LASCPC‐01 cells. d,e) IF staining and median fluorescence intensity (MFI) quantification showing the upregulated O‐GalNAc glycosylation in *rb1* ^Δ/Δ^ *p53* ^Δ/Δ^ NEPC organoids compared to *myc* ^hi^ *pten* ^Δ/Δ^ PrAD counterparts (scale bar = 20 µm). f–g) IF staining and MFI quantification showing the upregulated O‐GalNAc glycosylation in *rb1* ^Δ/Δ^ *p53* ^Δ/Δ^ NEPC organoids compared to *myc* ^hi^ *pten* ^Δ/Δ^ PrAD counterparts (scale bar = 10 µm). h–j) O‐GalNAc inhibitor Benzyl‐α‐GalNAc (5 µm, treated for 24 h) significantly suppressed the MBL binding and activation capabilities of *rb1* ^Δ/Δ^ *p53* ^Δ/Δ^ NEPC organoids. k,l) IF staining and MFI quantification showing the upregulated O‐GalNAc glycosylation in *rb1* ^Δ/Δ^ *p53* ^Δ/Δ^‐scramble and *rb1* ^Δ/Δ^ *p53* ^Δ/Δ^‐shGalnt9 NEPC organoids (scale bar = 50 µm). m–o) *Galnt9*‐KD in *rb1* ^Δ/Δ^ *p53* ^Δ/Δ^ NEPC organoids resulted in significantly decreased MBL binding (m‐n) and activation capabilities. p–q) IF staining images and MFI quantification revealed the O‐GalNAc glycosylation status in LAPC4, VCaP, and LASCPC‐01 cells (scale bar = 10 µm). r–t) *GALNT9*‐KD in human NEPC LASCPC‐01 cells resulted in significantly decreased MBL binding and activation capabilities. u–w) Dissected livers, H&E staining images, and quantification of the liver metastatic foci number w) of the C57BL/6 recipients inoculated with *rb1* ^Δ/Δ^ *p53* ^Δ/Δ^‐scramble (n = 7, mice) and *rb1* ^Δ/Δ^ *p53* ^Δ/Δ^‐sh*Galnt9* organoids (n = 8, mice). For statistics in this figure, the two‐tail unpaired Student's‐*t* test was applied for (e), (g), (i‐j) and (w), and the one‐way ANOVA test was applied for (n‐o), (q), and (s‐t). Data were shown as means ± SD.

Glycosylation is a complex process that encompasses a series of enzymatic reactions, which can be further divided into distinct stages. These stages include initiation, core extension, elongation, branching, and capping.^[^ ^19^ ^]^ The initial two stages are of particular specificity and significance.^[^ ^20^ ^]^ Therefore, we focused our attention on enzymes involved in the initiation and core extension steps of glycosylation to identify those that are upregulated in NEPC and crucial for synthesizing the glycans essential for MBL binding. Based on Beltran human PCa dataset,^[^ ^18^ ^]^ volcano plots revealed that the glycosyltransferase GALNT9 (encoded by *GALNT9*), which catalyzes O‐GalNAc glycosylation by adding the glycan of GalNAc to substrates, was most significantly upregulated in patients with NEPC compared with patients with PrAD (**Figure** **4**a). Consistently, significantly greater GALNT9 expression in NEPC than in PrAD from the SU2C PCa dataset was also identified (Figure S4a, Supporting Information).

To validate this finding, we assessed the expression level of GALNT9 in PrAD and NEPC samples from mouse models and/or human cell lines. As shown in Figure 4b, immunoblotting data revealed that Galnt9 protein expression was prominently elevated in the *rb1* ^Δ/Δ^ *p53* ^Δ/Δ^ NEPC organoids compared with the *myc* ^hi^ *pten* ^Δ/Δ^ PrAD and WT prostate organoids. Similarly, the human NEPC cell line LASCPC‐01 also presented much greater GALNT9 expression than did the human PrAD cell lines, including the androgen‐dependent VCaP, LNCaP, and 22Rv1 cell lines and the androgen‐independent Du145 and PC3 cell lines (Figure 4c). We then utilized multiple assays to assess the O‐GalNAc levels in murine (Figure 4d–g) and human (Figure S4b–d) NEPC versus PrAD samples. Immunofluorescence (IF) images revealed that murine *rb1* ^Δ/Δ^ *p53* ^Δ/Δ^ NEPC organoids presented significantly higher levels of O‐GalNAc than their *myc* ^hi^ *pten* ^Δ/Δ^ PrAD counterparts did (Figure 4d,e). Immunohistochemical (IHC) staining confirmed that both GALNT9 and O‐GalNAc were increased in clinical biospecimens from patients with NEPC compared with those from patients with PrAD (Figure S4b–d, Supporting Information).

To verify whether these findings in NEPC can be applied to other NE cancers, we examined GALNT9 and O‐GalNAc levels in the lung adenocarcinoma (LUAD) cell line A549 and the small cell lung cancer (SCLC) cell lines NCI‐H146 and NCI‐H82. Immunoblotting data (Figure S5a, Supporting Information) and IF staining (Figure S5b,c, Supporting Information) confirmed that both GALN9 and O‐GalNAc were significantly elevated in SCLC compared with LUAD. Notably, the upregulated O‐glycosylation was most prominently detected on the surface of NE cancer cells.

To determine the role of O‐GalNAc in MBL binding and activation in NEPC, an O‐GalNAc inhibitor, benzyl‐α‐GalNAC, was used to treat murine *rb1* ^Δ/Δ^ *p53* ^Δ/Δ^ NEPC organoids. IF staining (Figure 4f,g) confirmed that O‐GalNAc was evidently decreased following benzyl‐α‐GalNAc treatment. In addition, significant reductions in MBL binding and activation were observed in the *rb1* ^Δ/Δ^ *p53* ^Δ/Δ^ organoids following benzyl‐α‐GalNAC treatment (Figure 4h–j). We then established two lines of *Galnt9*‐knockdown (Galnt9‐KD) *rb1* ^Δ/Δ^ *p53* ^Δ/Δ^ NEPC organoids (shGalnt9‐1# and 2#) (Figure S6a, Supporting Information). IF staining demonstrated that Galnt9‐KD in the *rb1* ^Δ/Δ^ *p53* ^Δ/Δ^ NEPC organoids led to a significant reduction in the number of O‐GalNAc glycans on the cell membrane surface (Figure 4k,l). As shown in Figure 4m–o, the MBL binding and activation in the *rb1* ^Δ/Δ^ *p53* ^Δ/Δ^ organoids were significantly repressed following *Galnt9* knockdown. Similarly, IF staining demonstrated that O‐GalNAc levels were significantly greater in the human NEPC cell line LASCPC‐01 than in the PrAD cell lines VCaP and LAPC4 (Figure 4p,q). Compared with scramble control cells, LASCPC‐01‐shGALNT9 cells (Figure S6b, Supporting Information) also presented a significant reduction in MBL binding/activation (Figure 4r–t). We performed additional in vivo experiments in which either the *rb1* ^Δ/Δ^ *p53* ^Δ/Δ^‐shGalnt9 organoids or scrambled controls were injected intravenously into WT mice. As shown in Figure 4u–w, mice inoculated with *rb1* ^Δ/Δ^ *p53* ^Δ/Δ^‐shGalnt9 organoids presented a decreased liver metastatic burden.

Next, we utilized the SCLC cell line NCI‐H146 (H146) and the neuroendocrine colorectal cancer cell line COLO‐320DM (COLO) to assess the generalizability of these findings in NEPC to a broader range of NE cancers. Intriguingly, knockdown of *GALNT9* in both NCI‐H146 (Figure S6c, Supporting Information) and COLO‐320DM cells (Figure S6d, Supporting Information) resulted in similarly significant reductions in MBL binding/activation in vitro (Figure S7a–f, Supporting Information). Furthermore, in vivo, experiments were conducted in which either COLO‐320DM‐shGALNT9 cells or control cells were injected intravenously into nude mice. As shown in Figure S7g–i (Supporting Information), mice inoculated with COLO‐320DM‐shGALNT9 cells exhibited a decrease in liver metastatic lesions. In summary, the expression of GALNT9 in NEPC, SCLC, and neuroendocrine colon cancers is essential for liver metastasis.

**Figure 5:**
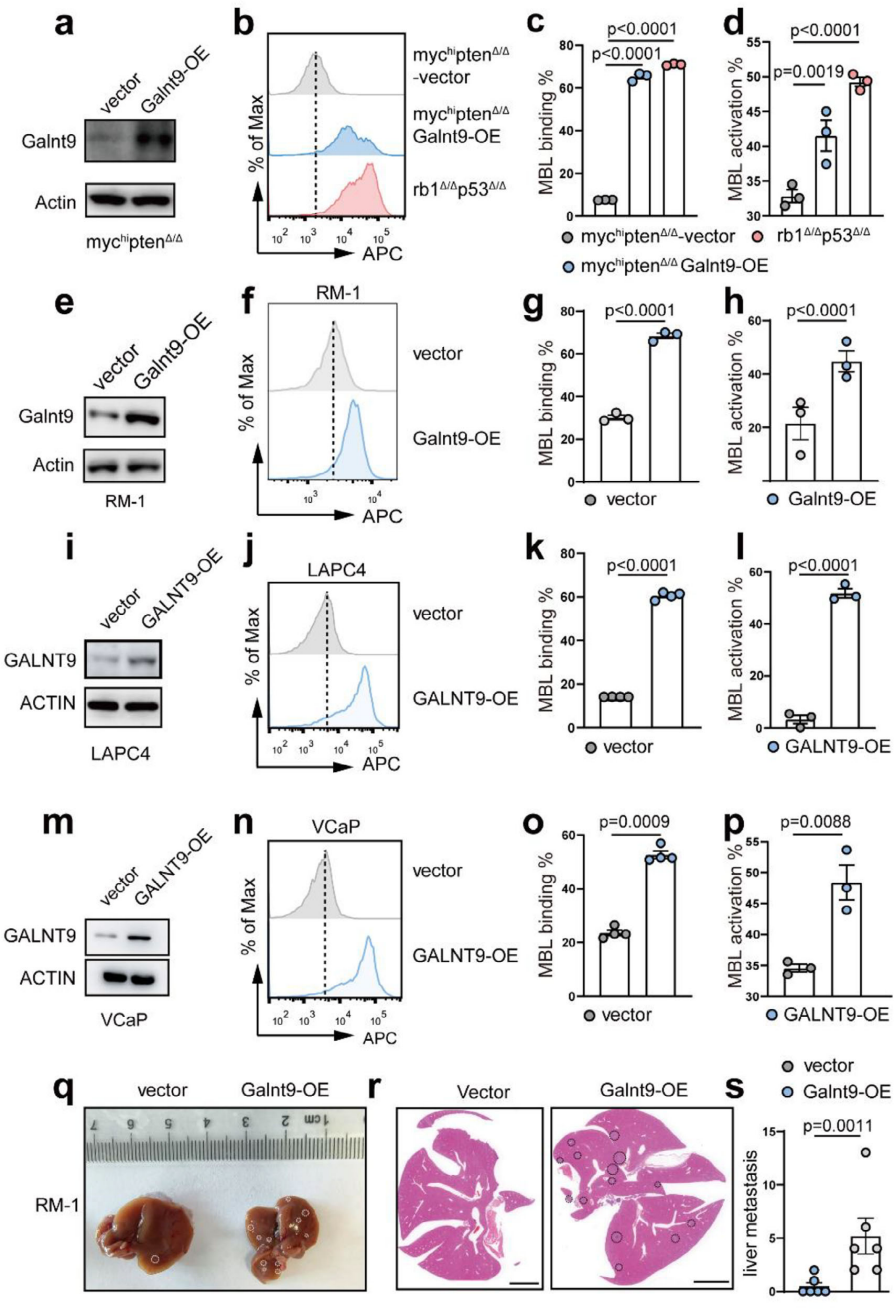
Galnt9‐OE in PrAD enhances MBL activation and promotes liver metastasis. a) Immunoblotting assay confirming the ectopic expression of *Galnt9* (Galnt9‐OE) in *myc* ^hi^ *pten* ^Δ/Δ^ PrAD organoids. b–d) Galnt9‐OE in *myc* ^hi^ *pten* ^Δ/Δ^ PrAD organoids significantly increased MBL binding and activation capabilities. e) Immunoblotting assay showing that Galnt9 is overexpressed in murine RM‐1 PrAD cells. f–h) Galnt9‐OE in murine RM‐1 PrAD cells significantly increased MBL binding and activation capabilities. i) Immunoblotting assay confirming the ectopic expression of *GALNT9* (GALNT9‐OE) in human LAPC4 PrAD cells. j–l) GALNT9‐OE in human LAPC4 PrAD cells significantly increased MBL binding j,k) and activation l) capabilities. m) Immunoblotting assay confirming the ectopic expression of *GALNT9* (GALNT9‐OE) in human VCaP PrAD cells. n–p) GALNT9‐OE in human VCaP PrAD cells significantly increased MBL binding and activation p) capabilities. q–s) Dissected livers, H&E staining images, and quantification of the liver metastatic foci number of the C57BL/6 recipients intravenously inoculated with RM‐1‐vector and RM‐1‐Galnt9‐OE cells (n = 6, mice). For statistics in this figure, the two‐tail unpaired Student's‐*t* test was applied for (g‐h), (k‐l), (o‐p) and (s), and the one‐way ANOVA test was applied for (c‐d). Data were shown as means ± SD.

We also asked whether ectopic expression of GALNT9 (GALNT9‐OE) in PrAD could enhance MBL binding/activation. To this end, we established a Galnt9‐OE murine *myc* ^hi^ *pten* ^Δ/Δ^ PrAD organoid line and found that it exhibited significantly stronger MBL binding and activation activities than did the empty vector‐transfected controls (**Figure** **5**a–d), although the MBL binding and activation activities were still lower than those of the *rb1* ^Δ/Δ^ *p53* ^Δ/Δ^ NEPC organoid line (Figure 5c–d). We also ectopically expressed Galnt9 in RM‐1 cells, another murine PrAD cell line (Figure 5e). Consistently, Galnt9‐OE in RM‐1 cells led to increased MBL binding and activation (Figure 5f–h). Next, we sought to validate these findings in human PCa contexts. GALNT9‐OE in two human PrAD cell lines, LAPC4 and VCaP, also led to increases in MBL binding and activation compared with the respective controls (Figure 5i–p). In addition to PCa, we ectopically overexpressed GALNT9 in the LUAD cell line A549 and found significantly elevated MBL binding and activation levels upon GALNT9‐OE (Figure S8a–d, Supporting Information). Collectively, our results suggest that GALNT9‐mediated O‐GalNAc modification is crucial for MBL binding and complement activation across a variety of NE cancers.

We next conducted in vivo experiments in which RM‐1‐Galnt9‐OE and RM‐1‐vector cells were intravenously implanted into C57BL/6 mice. Strikingly, compared with the vector control, Galnt9‐OE in PrAD cells induced significant liver metastasis (Figure 5q–s), highlighting the role of Galnt9 in promoting NEPC liver metastasis.

### 2.5 The Glycosylation‐Triggered MBL Pathway Promotes Platelet Activation and Facilitates NEPC Liver Metastasis

**Figure 6:**
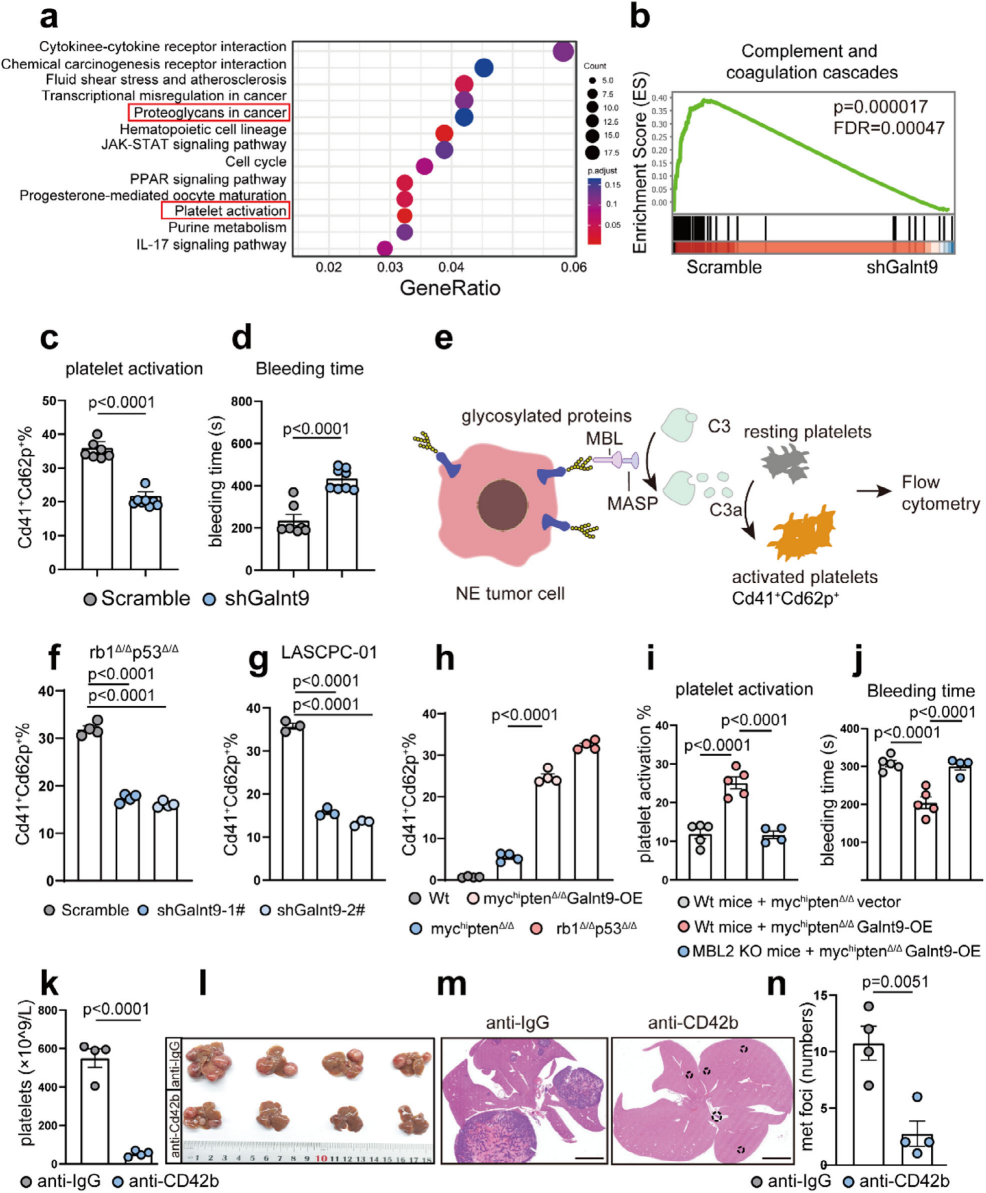
Glycosylation‐triggered MBL pathway promotes platelet activation and facilitates NEPC liver metastasis. a) The KEGG analysis showing that the “proteoglycans in cancer” and “platelet activation” were upregulated in *rb1* ^Δ/Δ^ *p53* ^Δ/Δ^‐scramble organoids versus *rb1* ^Δ/Δ^ *p53* ^Δ/Δ^‐sh*Galnt9* organoid counterparts. b) The GSEA plot reveals that the “complement and coagulation cascades” hallmark genes were significantly enriched in *rb1* ^Δ/Δ^ *p53* ^Δ/Δ^‐scramble organoids in comparison to *rb1* ^Δ/Δ^ *p53* ^Δ/Δ^‐sh*Galnt9* organoid counterparts. c) The in vivo platelet activation assay reveals that C57BL/6 recipients inoculated with *rb1* ^Δ/Δ^ *p53* ^Δ/Δ^‐shGalnt9 organoids (n = 8, mice) showed significantly decreased platelet activation capability in comparison to the mice‐receiving *rb1* ^Δ/Δ^ *p53* ^Δ/Δ^‐scramble organoids (n = 7, mice). d) The tail vein bleeding assay shows that C57BL/6 recipients inoculated with *rb1* ^Δ/Δ^ *p53* ^Δ/Δ^‐shGalnt9 organoids (n = 8, mice) takes significantly longer time in coagulation compared to the mice‐receiving *rb1* ^Δ/Δ^ *p53* ^Δ/Δ^‐scramble organoids (n = 7, mice). e) A schematic diagram of experimental design for platelet activation assay. f) Galnt9‐KD in *rb1* ^Δ/Δ^ *p53* ^Δ/Δ^ organoids leads to significantly deduced platelet activation capability compared to scramble control *rb1* ^Δ/Δ^ *p53* ^Δ/Δ^ organoids in vitro. g) GALNT9‐KD in human NEPC LASCPC‐01 cells leads to significantly reduced platelet activation capability compared to scramble control shRNA‐transfected LASCPC‐01 cells in vitro. h) Galnt9‐OE in murine *myc* ^hi^ *pten* ^Δ/Δ^ PrAD organoids significantly increased platelet activation capability compared to vector control PrAD organoids. i) Galnt9‐OE‐induced platelet activation in PrAD was diminished in MBL2‐KO recipients versus WT recipient counterparts (n = 5 mice). j) Galnt9‐OE‐mediated coagulation, as revealed by shorter bleeding time, in PrAD is reversed in MBL2‐KO recipients versus WT recipient counterparts (n = 5 mice). k–n) Depletion of platelet in vivo using anti‐CD42b antibody significantly attenuates the liver metastatic burdens in *rb1* ^Δ/Δ^ *p53* ^Δ/Δ^ organoid‐inoculated mice, as revealed by reduced liver metastatic foci numbers in contrast to anti‐IgG control treated tumor‐bearing mice (n = 4, mice). Scale bar = 5 mm in (m). For statistics in this figure, the two‐tail unpaired Student's‐*t* test was applied for (c‐d), (k) and (n), and the one‐way ANOVA test was applied for (f‐i). Data were shown as means ± SD.

Complement activation and the coagulation cascade are closely intertwined biological processes.^[^ ^21^ ^]^ Considering that thrombi coexist with NEPC liver metastasis and that glycosylation in NEPC triggers the MBL pathway for complement activation, we asked whether platelet activation is a downstream effect and a necessary step in promoting NEPC liver metastasis. To do this, we isolated *rb1* ^Δ/Δ^ *p53* ^Δ/Δ^‐scramble and *rb1* ^Δ/Δ^ *p53* ^Δ/Δ^‐Galnt9‐KD NEPC liver metastatic foci for bulk RNA‐seq. As shown in **Figure** **6**a, two glycosylation‐associated hallmarks, the “proteoglycans in cancer” and “platelet activation” pathways, were significantly enriched in *rb1* ^Δ/Δ^ *p53* ^Δ/Δ^‐scramble liver metastases compared with their Galnt9‐KD counterparts. In support of these data, GSEA plots revealed a significant downregulation of the “complement and coagulation cascades” signature in *rb1* ^Δ/Δ^ *p53* ^Δ/Δ^‐Galnt9‐KD liver metastases (Figure 6b), suggesting that *rb1* ^Δ/Δ^ *p53* ^Δ/Δ^ organoids expressing Galnt9 may incur increased susceptibility to thrombosis and platelet activation when engrafted within murine hosts. To test this hypothesis, we collected peripheral blood from *rb1* ^Δ/Δ^ *p53* ^Δ/Δ^‐scramble and *rb1* ^Δ/Δ^ *p53* ^Δ/Δ^‐Galnt9‐KD tumor‐bearing mice to evaluate their platelet activation status (Figure 6c). We found a significant decrease in the number of Cd41^+^Cd62p^+^‐activated platelets in the peripheral blood of the *rb1* ^Δ/Δ^ *p53* ^Δ/Δ^‐Galnt9‐KD organoid‐xenografted mice (Figure 6c). In line with these findings, vein bleeding tests revealed that *rb1* ^Δ/Δ^ *p53* ^Δ/Δ^‐Galnt9‐KD tumor‐bearing mice took a significantly longer time to coagulate (Figure 6d).

Next, we performed an in vitro platelet activation assay (Figure 6e). In this study, cancer cells were incubated with MBL, MASPs, and complement C3 for complement activation, and the supernatant was collected to treat murine platelets. Platelet activation was evaluated by flow cytometry, and the Cd41^+^Cd62p^+^ cell population was calculated. Galnt9‐KD in both mouse *rb1* ^Δ/Δ^ *p53* ^Δ/Δ^ NEPC organoids and the human NEPC cell line LASCPC‐01 led to significantly attenuated platelet activation in vitro (Figure 6f,g; Figure S9a,b, Supporting Information). Therefore, our data suggest that elevated expression of GALNT9 in NEPC promotes platelet activation.

Furthermore, we found that in vitro platelet activation in *myc* ^hi^ *pten* ^Δ/Δ^ Galnt9‐OE organoids was significantly increased, although it was still lower than that in *rb1* ^Δ/Δ^ *p53* ^Δ/Δ^ NEPC organoids (Figure 6h; Figure S9c, Supporting Information). In addition, we intravenously injected *myc^hi^pten^Δ/Δ^*‐vector and *myc^hi^pten^Δ/Δ^*‐Galnt9‐OE organoids into WT and MBL2‐KO mice and assessed their platelet activation status and coagulation time. An increase in the Cd41+Cd62p+‐activated platelet population and a shortened bleeding time were observed in the *myc^hi^pten^Δ/Δ^*‐Galnt9‐OE group (Figure 6i,j). Notably, these effects were abrogated by MBL2 knockout (Figure 6i,j).

To further investigate the role of platelet activation in promoting NEPC liver metastasis in vivo, we depleted platelets using an anti‐CD42b blocking antibody in *rb1* ^Δ/Δ^ *p53* ^Δ/Δ^‐NEPC organoid‐inoculated mice (Figure 6k). Compared with IgG, the anti‐CD42b antibody significantly suppressed the liver metastatic burden in NEPC tumor‐bearing mice (Figure 6l–n), indicating that platelet activation facilitates NEPC liver metastasis. Taking together, our data suggest that O‐GalNAc catalyzed by Galnt9 triggers the activation of the MBL complement pathway, leading to platelet activation and liver metastasis, with MBL serving as an essential component of this process.

### 2.6 Inhibition of O‐Glycosylation with a GalNAc Inhibitor Blocks NE Cancer Liver Metastasis

**Figure 7:**
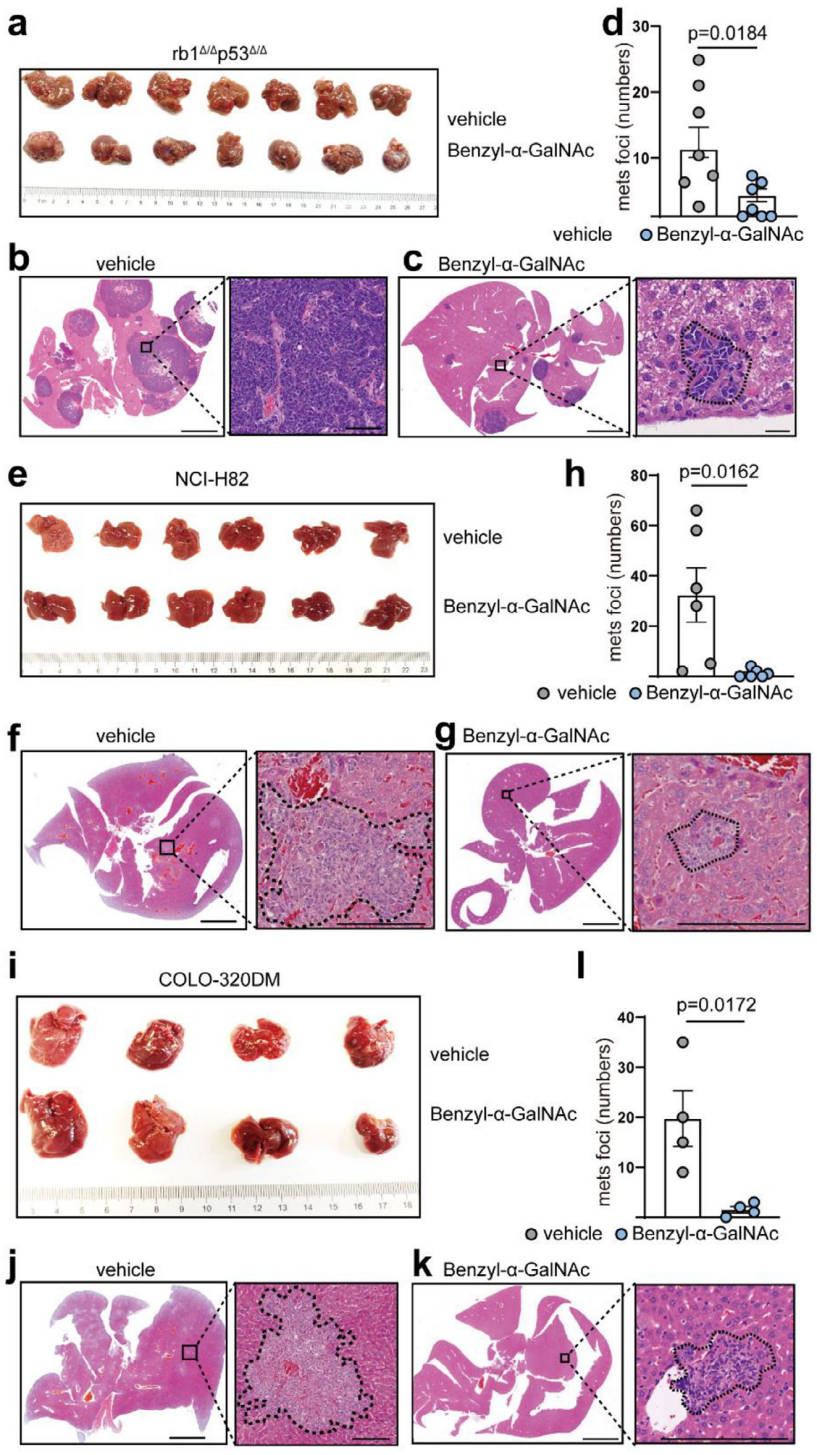
Blockade of O‐GalNAc suppresses liver metastasis in a wide range of NE cancers. a–d) The O‐GalNAc inhibitor benzyl‐α‐GalNAc treatment leads to significantly reduced liver metastatic burdens in *rb1* ^Δ/Δ^ *p53* ^Δ/Δ^ organoid‐inoculated C57BL/6 mice (n = 7), as exemplified by representative H&E staining imagesscale bar = 5 mm) and quantification data. e‐h) Benzyl‐α‐GalNAc treatment results in significantly attenuated liver metastatic burdens in SCLC NCI‐H82‐inoculated nude mice (*n* = 6), as revealed by representative H&E staining images, scale bar = 5 mm) and quantification data. i–l) Benzyl‐α‐GalNAc treatment leads to significantly repressed liver metastatic lesions in neuroendocrine colon cancer cell COLO‐320DM inoculated nude mice (*n* = 4), as exemplified by representative H&E staining images, scale bar = 5 mm) and quantification results. For statistics in (d), (h), and (l) student's t‐test was applied and data were shown as mean ± SD. P value ≤ 0.01 was considered as statistically significant. For statistics in this figure, the two‐tail unpaired Student's‐*t* test was applied for (d), (h) and (l). Data were shown as means ± SD.

We evaluated the impact of O‐GalNAc glycosylation inhibition on liver metastasis in NEPC and other NE cancers. Following intraperitoneal (i.p.) administration of the GalNAc inhibitor benzyl‐α‐GalNAc, we observed a significant reduction in the liver metastasis burden (**Figure** **7**a‐d) in the *rb1^Δ/Δ^p53^Δ/Δ^* organoid‐inoculated mice. We then tested the effects of benzyl‐α‐GalNAc in other NE cancer types, including SCLC NCI‐H82 (H82) cells (Figure 7e–h) and neuroendocrine colon cancer COLO‐320DM cells (Figure 7i–l). Significant decreases in liver metastasis were observed in mice bearing NCI‐H82 or COLO‐320DM tumors following benzyl‐α‐GalNAc treatment (Figure 7e–l). These findings demonstrate the therapeutic potential of O‐GalNAc glycosylation inhibition in the treatment of liver metastasis in a wide range of NE carcinomas.

### 2.7 O‐Glycosylated ANXA2 is a Key Protein that Binds to and Activates the MBL2–MASP Complement Pathway in NEPC

Both protein and RNA utilize the same enzymatic machinery to mediate O‐glycosylation. Recent studies have shown that glycosylated RNA on the surface of neutrophils enhances neutrophil recruitment, adhesion, and migration.^[^ ^22^ ^]^ We sought to determine whether glycosylated proteins or RNAs play a primary role in the activation process of the MBL complement pathway in NE cancers. An MBL binding assay was conducted in *rb1* ^Δ/Δ^ *p53* ^Δ/Δ^ NEPC organoids, with the addition of exogenous RNase to remove extracellular RNA or proteinase K to degrade extracellular proteins. Our findings revealed a pronounced reduction in MBL binding following the digestion of extracellular proteins but not RNA (Figure S10a,b, Supporting Information). Considering these findings, our research focused on glycosylated proteins.

**Figure 8:**
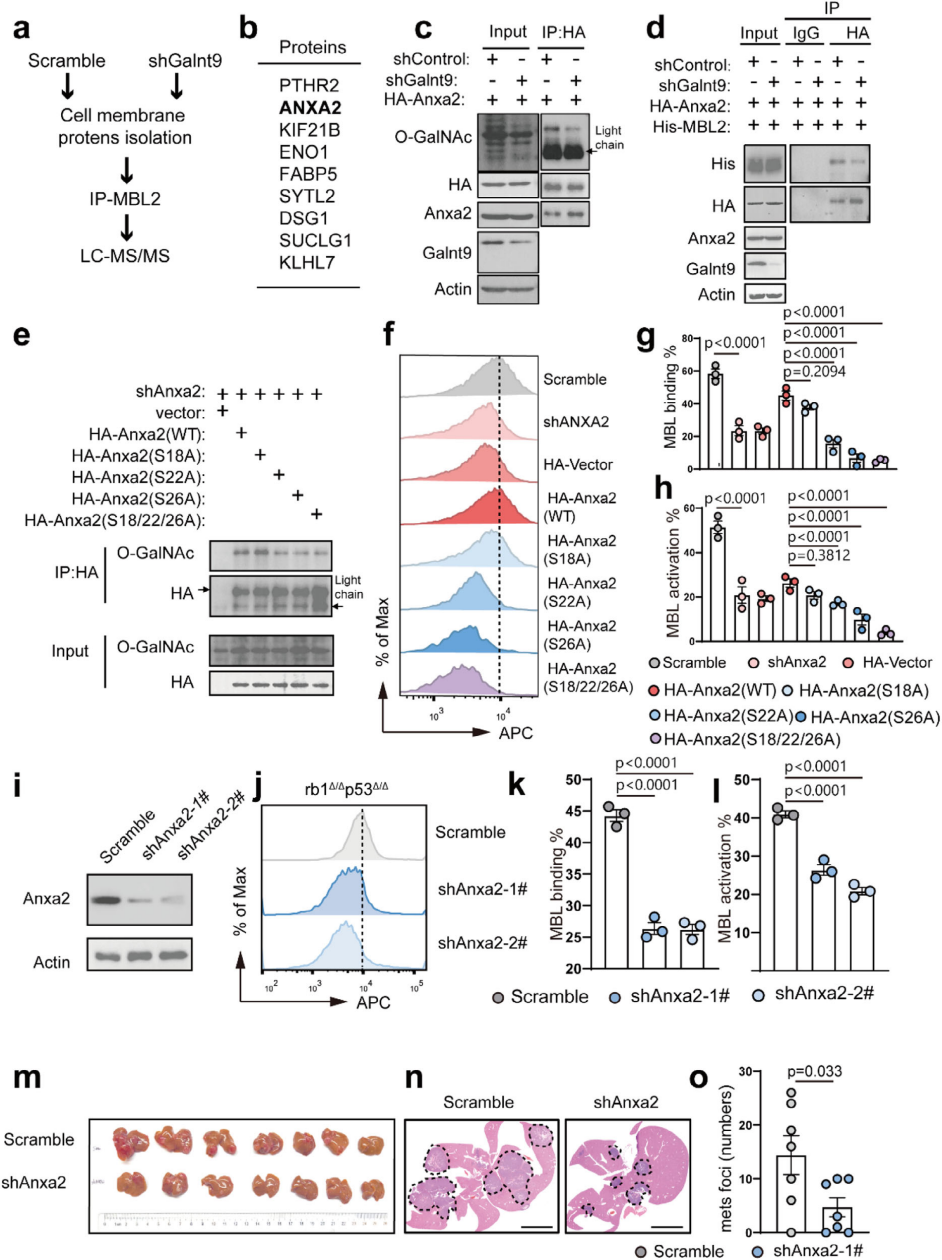
O‐glycosylated ANXA2 is a key protein that binds to and activates the MBL complement pathway in NEPC. a) Schematic diagrams illustrating the workflow for screening Galnt9 substrates using immunoprecipitation (IP) and mass spectrometry (MS). b) MS screening data showing a candidate list of Galnt9 substrates. c) Immunoprecipitation (IP) assay showing that Galnt9‐KD attenuates the O‐GalNAc glycosylation in murine *rb1* ^Δ/Δ^ *p53* ^Δ/Δ^ NEPC cells. d) Immunoprecipitation (IP) assay demonstrates that Galnt9‐KD impairs the interaction between Anxa2 and MBL2. e) Immunoprecipitation (IP) assay demonstrates the mutations of the key O‐GalNAc glycosylated sites of Anxa2 including the 18th, 22nd, and 26th Serine residues (S18A, S22A, S26A) impaired O‐Glycosylation levels as compared to the intact WT Anxa2 construct. f–h) Mutations of *Anxa2* in the 22nd and 26th serine sites result in significantly reduced MBL binding and activation capabilities. i‐l) Anxa2‐KD in *rb1* ^Δ/Δ^ *p53* ^Δ/Δ^ NEPC organoids leads to significantly reduced MBL binding j,k) and activation l) capabilities as compared to scramble controls. m–o) Dissected livers, H&E staining images, and quantification results on liver metastatic foci number of the C57BL/6 recipients inoculated with *rb1* ^Δ/Δ^ *p53* ^Δ/Δ^‐scramble organoids and *rb1* ^Δ/Δ^ *p53* ^Δ/Δ^‐shAnxa2 organoids (n = 7, mice). For statistics in this figure, the two‐tail unpaired Student's‐*t* test was applied for (o), and the one‐way ANOVA test was applied for (g‐h) and (k‐l). Data were shown as means ± SD.

To explore the key surface O‐glycosylated proteins on NE cancer cells that bind and activate MBL signaling, the cell membrane proteins of the *rb1* ^Δ/Δ^ *p53* ^Δ/Δ^ Galnt9‐KD‐NEPC organoids and *rb1* ^Δ/Δ^ *p53* ^Δ/Δ^ scramble counterparts were fractionated and incubated with purified recombinant His‐tagged MBL2 protein in vitro (**Figure** **8**a). The MBL2‐associated proteins were then immunoprecipitated (IP) with an anti‐His antibody and subsequently subjected to mass spectrometry (Figure 8a). Thus, we identified a panel of MBL2‐associated target proteins that rely on Galnt9 (Figure 8b). Among them, the cell membrane adhesion molecule Annexin A2 (encoded by *ANXA2*), which has been reported to predict a significantly unfavorable clinical outcome in patients with PCa,^[^ ^23^ ^]^ has attracted our attention (Figure 8b). In the human PCa databases of SU2C and Beltran, ANXA2 was significantly higher in patients with NEPC than in patients with PrAD (Figure S11a,b, Supporting Information). To validate these data, we collected three sets of NEPC and PrAD clinical biospecimens and performed immunoblotting assays. As shown in Figure S11c (Supporting Information), human NEPC tissues presented much higher ANXA2 levels than their human PrAD counterparts did.

We also observed that Galnt9‐KD in *rb1* ^Δ/Δ^ *p53* ^Δ/Δ^ organoids resulted in a significant reduction in the O‐GalNAc level of Anxa2 (Figure 8c). Co‐IP experiments confirmed the interaction between Anxa2 and MBL2 in murine NEPC *rb1* ^Δ/Δ^ *p53* ^Δ/Δ^ organoids, and the physical association between Anxa2 and MBL2 was impaired by Galnt9 KD, suggesting an essential role of Galnt9‐mediated Anxa2 O‐GalNAc modification in the physical interaction between Anxa2 and MBL2 (Figure 8d).

Using an online O‐GalNAc glycosylation prediction tool (NetOGlyc‐4.0),^[^ ^24^ ^]^ three amino acid residues were identified, including the 18th, 22nd, and 26th serine residues (S18, S22, and S26) of Anxa2, as potential O‐GalNAc modification sites. We constructed HA‐tagged Anxa2 plasmids, including WT and mutant forms: Anxa2(S18A), Anxa2(S22A), Anxa2(S26A), and Anxa2(S18,22,26A). The *rb1* ^Δ/Δ^ *p53* ^Δ/Δ^‐shAnxa2 NEPC organoids were used for transfection as a clean system. As shown in Figure 8e, the O‐GalNAc level of Anxa2 decreased markedly upon Anxa2(S22A) and (S26A) transfections, suggesting that these two serine sites on Anxa2 are required for its O‐GalNAc modification.

To further determine the functional importance of ANXA2's O‐GalNAc in binding and activating MBL and promoting NEPC liver metastasis, the *rb1* ^Δ/Δ^ *p53* ^Δ/Δ^ NEPC cells were utilized that expressing mutant Anxa2 constructs with shAnxa2 transfection for in vitro and in vivo experiments. As shown in Figure 8f–h, mutant sites in S22A and S26A of Anxa2 showed significantly reduced MBL binding and activation in comparison to WT Anxa2. In addition, Anxa2‐KD in *rb1* ^Δ/Δ^ *p53* ^Δ/Δ^ NEPC cells (Figure 8i) also incurred significant repression of MBL binding (Figure 8j,k) and activation (Figure 8l). Finally, the *rb1* ^Δ/Δ^ *p53* ^Δ/Δ^‐shAnxa2 and *rb1* ^Δ/Δ^ *p53* ^Δ/Δ^‐scramble organoids were intravenously inoculated into C57BL/6 recipients, respectively. As shown in Figure 8m–o, knockdown of *Anxa2* in NEPC cells led to significantly attenuated liver metastatic burdens. Therefore, our data suggested that Galnt9‐catalyzed O‐GalNAc glycosylation on Anxa2 plays a crucial role in driving NEPC liver metastasis in dependence on the MBL complement activation signaling.

## 3 Discussion

In this study, we find that thrombosis is a common occurrence in NEPC liver metastatic foci and that the complement and coagulation cascades are activated in liver metastasis. Mechanistically, our study demonstrates that the high levels of O‐GalNAc modifications on NEPC cell surface proteins, particularly ANXA2, catalyzed by the glycosyltransferase GALNT9, bind and activate the MBL complement pathway. This triggers platelet activation and thrombus formation, promoting hepatic metastasis. Targeting of GALNT9 or O‐GalNAc glycosylation can effectively inhibit the development of liver metastasis in NE cancers (Figure S12, Supporting Information, Graphical abstract).

The major upstream cues responsible for complement activation within the tumor microenvironment (TME) remain unclear, although it has long been recognized that the complement system plays a crucial role in tumor development and progression.^[^ ^25^ ^]^ In a cervical cancer model, antigen‐antibody complexes within the TME have been observed to activate the classical complement pathway.^[^ ^26^ ^]^ However, prior research was unaware that cancer cells per se might activate the complement system as well. The present study demonstrates that the glycosylation on the surface of NE cancer cells can activate the complement system through the MBL‐MASPs pathway. Among various glycosyltransferases, Galnt9 is of particular significance in NE cancers. The correlation between Galnt9 and the clinical prognosis of neuroblastoma^[^ ^27^ ^]^ and serous ovarian cancer^[^ ^28^ ^]^ has been well‐established, and its association with brain metastasis in breast cancer^[^ ^29^ ^]^ has also been documented. However, the role and underlying mechanism of GLANT9 in cancer progression have not been identified. Conventionally, the MBL pathway is activated upon pathogen invasion due to glycosylated surface. Our findings indicate that NE cancer cells mimic this pathophysiological process to trigger MBL signaling and complement activation via a distinct form of glycan, the GALNT9‐catalyzed O‐GalNAc. Therefore, GALNT9 plays a pivotal role in driving liver metastasis of NE cancers in the liver by adding O‐GalNAc glycans to cell surface proteins. High mannose has been well established as a key glycan involved in binding and activating MBLs.^[^ ^14^ ^]^ However, we demonstrate here that O‐GalNAc on NEPC cells binds to MBL2 and activates the complement system via the lectin pathway. This finding broadens our understanding of the MBL recognition mechanism.

Apart from the intrinsic characteristics of cancer cells, the liver microenvironment exerts a substantial influence on cancer metastasis. Hepatocytes are the primary cells that produce coagulation factors, and the liver is a major organ to generates complement proteins in the human body.^[^ ^30^ ^]^ This makes the liver a vulnerable site to initiate complement activation and coagulation cascade reactions. In the current study, we demonstrate that NE cancer cells express high levels of glycoproteins, enabling effective activation of the MBL pathway and platelets within the liver microenvironment. Additionally, the liver is characterized by an abundance of blood supply and hepatic sinusoid.^[^ ^31^ ^]^ Coagulation and thrombus formation result in a reduction in blood flow rate and facilitate cancer cell attachment and colonization.^[^ ^32^ ^]^ Therefore, the liver microenvironment and the characteristics of NE cancer cells collectively facilitate the colonization of cancer cells in the liver. The findings of our study provide a deeper understanding of the mechanisms underlying the organotropic metastasis of NE cancer to the liver.

Liver metastasis of NE cancers is a significant clinical challenge with a poor prognosis and a dearth of efficacious therapeutic options. Despite the distinct anatomical origins and histopathological divergence among various NE cancers, GALNT9‐mediated ANXA2 O‐GalNAc acts as a pan‐cancer mechanism to trigger MBL complement activation, coagulation, and thrombus formation, thus promoting liver metastasis of NE malignancies, including NEPC, SCLC, and neuroendocrine colon cancer. Targeting this pathway holds promise as a strategy for developing therapeutic interventions against liver metastasis in NE carcinomas.

## 4 Experimental Section

### Mice and Organoids

All genetically engineered mouse models (GEMMs) and tumor organoid‐ingrafted mice in this study were housed in specific pathogen‐free (SPF) facilities at the Animal Research Center of Renji Hospital. All animal experiments were approved by the Animal Use and Care Committee of Renji Hospital, Shanghai Jiao Tong University School of Medicine (RJ2022‐0711). The prostate‐specific dual depletion of *rb1* and *p53* (*rb1^Δ/Δ^p53^Δ/Δ^*) NEPC‐like organoids were obtained by crossing the Probasin‐Cre (PbCre) transgenic mice with the lines, in which, the LoxP cassette was inserted into interested gene locus. The prostate‐specific ectopic expression of *Myc* and ablation of *Pten* transgenic mice (*myc* ^Hi^ *Pten* ^Δ/Δ^), in which, their prostate tumors phenocopy prostate adenocarcinoma (PrAD) features, were obtained from the Jackson laboratory. The *rb1^Δ/Δ^p53^Δ/Δ^* NEPC and *myc* ^Hi^ *Pten* ^Δ/Δ^ PrAD murine prostate cancer organoid lines were established and cultured as it was previously reported.

The whole‐body mannose binding lectin 2 (MBL2)‐knockout (*MBL2* ^‐/‐^, MBL2‐KO) mice were commercially available from GemPhamatech Co. Ltd (Jiangsu, China). For tumor organoid and cell line allograft and/or xenograft assays, C57BL/6 mice (male, 6–8 week‐old) and/or athymic nu/nu nude mice (male, 6–8 week‐old) were purchased from Shanghai SLAC Laboratory Animal Co. Ltd (Shanghai, China). For prostate orthotopic implantation assays, 10^5^ spheres of *rb1^Δ/Δ^p53^Δ/Δ^* NEPC murine organoids were counted and inoculated into the anterior lobe prostate of WT C57BL/6 mice. For intravenous inoculation assays, 10^6^ NCI‐H82, COLO‐320DM, or other indicated tumor cells were injected into the immune‐deficient nude mice. For administration of O‐GalNac inhibitor of Benzyl‐α‐GalNAc (MedChemExpress, 3554‐93‐6, 2.5 mg kg^−1^, dissolved in 200 mL PBS solution), the tumor‐bearing mice were intraperitoneally injected every 3 days and lasts for 3 weeks after tumor cell inoculation.

### Cell Lines

Human prostate cancer (PCa) cell lines used in this study include VCaP, and LASCPC‐01, which were purchased from American Type Culture Collection (ATCC). The PCa cell line LAPC4 was kindly provided from C. Sawyers (Memorial Sloan Kettering Cancer Center). Murine PCa cell lines include RM‐1, which was kindly provided from Dr. Qi. Wang at Ren Ji Hospital), and the *rb1^Δ/Δ^p53^Δ/Δ^* and *Myc* ^Hi^ *Pten* ^Δ/Δ^ organoid‐derived tumor cells cultured in 2D conditions. Human lung cancer cell lines included lung adenocarcinoma (LUAD) A549, small cell lung cancer (SCLC) NCI‐H146, and NCI‐H82, which were commercially available from ATCC. Human neuroendocrine colon cancer cell line COLO‐320DM was purchased from ATCC. All these cell lines were validated by short tandem repeat profiling (Shanghai Biowing Applied Biotechnology).

For cell cultures, the 22Rv1, VCaP, COLO‐320DM, LAPC4, Du145, and PC3 cells were maintained in Dulbecco's Modified Eagle Medium (DMEM) supplemented with 10% fetal bovine serum (FBS, Gibco) and 100×Penicillin‐Streptomycin solution (P/S, Gibco). The NCI‐H146, NCI‐H82, and A549 cells were cultured in Roswell Park Memorial Institute (RPMI) 1640 medium containing 10% FBS and P/S solution. The LASCPC1‐01 cells were maintained in RPMI‐1640 medium with 5% FBS, 100×P/S solution, 0.005 mg mL^−1^ Insulin (0.005 mg mL^−1^), Transferrin (0.01 mg mL^−1^), Sodium selenite (30 nm), 10 nm Hydrocortisone (10 nm), 10 nm β‐estradiol (10 nm), and 2 mm L‐glutamine (2 mm), These above‐mentioned supplements for LASCPC‐01 cells were purchased from Sigma–Aldrich Co. Ltd. Cells were maintained at 37 °C with 5% CO_2_ incubators. All cells utilized in this study were confirmed by short tandem repeat (STR) analyses.

### Mass Spectrometry Analysis of MBL2‐Associated Membrane Proteins

The MBL2‐associated membrane proteins were analyzed by Liquid Chromatography‐Tandem Mass Spectrometry (LC‐MS/MS, Shanghai Applied Protein Technology). Briefly, the isolated membrane protein lysates were incubated with the recombinant protein His‐MBL2 (Sino Biological, Cat 50063‐M07H), and MBL2‐interacting protein complex was immunoprecipitated followed with SDS‐PAGE and coomassie blue staining. The protein bands of interest were cut for analysis by LC‐MS/MS. The mass‐charge ratio of peptides and peptide fragments was acquired using 20 fragment profiles (MS2 scan) after each full scan, with an MS2 Activation Type of Higher‐energy collisional dissociation (HCD), an isolation window of 2 m z^−1^, a secondary mass resolution of 17 500 at 200 m z^−1^, a normalized collision energy of 27 eV, and an underfill of 0.1. The raw file of the mass spectrometry test was searched by the engine MaxQuant 1.6.14 to retrieve the corresponding database, and finally, the identified protein candidates were obtained.

### MBL Binding Assay

1×10^6^ *rb1* ^Δ/Δ^ *p53* ^Δ/Δ^ NEPC or *myc* ^hi^ *pten* ^Δ/Δ^ PrAD murine organoids or their 2D cultured tumor cell derivatives were prepared into single‐cell suspensions in 100 µL phosphate‐buffered saline (PBS). Next, NEPC or PrAD cells were incubated with 10 µg mL^−1^ of Mannan‐Binding Lectin 2 (MBL2) protein‐fused Fc domain (MBL2‐Fc) at 4 °C for 1 h. After that, tumor cells were washed with PBS to remove the unbound MBL2‐Fc proteins. Subsequently, the MBL2‐bound cells were added with 1 µL anti‐Fc‐APC‐conjugated antibody. Then, the MBL binding activity of NEPC and/or PrAD cells was measured by flow cytometric analyses via quantification of the APC positive cell proportion. For data in Figure 3, tumor cells were pretreated with α‐mannosidase (4 U µL^−1^) or O‐Glycosidase (4 U µL^−1^) at 4 °C for 1 h and followed by routine MBL binding assay.

### MBL Activation Assay

1 × 10^6^ *rb1* ^Δ/Δ^ *p53* ^Δ/Δ^ NEPC or *myc* ^hi^ *pten* ^Δ/Δ^ PrAD murine organoids or their 2D cultured tumor cell derivatives were prepared as single‐cell suspensions in 100 µL PBS‐containing reaction system. Then, 10 µg mL^−1^ of MBL2 and Mannose‐Binding Protein‐associated Serine Proteases (MASPs) were added into the 100 µL PBS reaction system and followed by 1 h incubation on ice. Subsequently, 50 µL C3 complement component (200 U mL^−1^) was added into each reaction system for 30 min incubation at 4 °C for 1 h. After that, tumor cells were centrifuged at 1, 000 rpm for 5 min, and the supernatant was collected. Next, 100 µL of sensitized sheep red blood cells (SRBCs) was added into the supernatant obtained from the last step for 15 min incubation at 37 °C. Upon complement activation, hemolysin induces the lysis of SRBCs. The MBL‐activation capability of cancer cells was assessed by measuring the SRBCs hemolytic degree reflected by the absorbance at OD_542_ using a spectrophotometry instrument.

### Lectin Microarray

Utilizing a commercial lectin microarray kit (RayBiotech, GA‐Lectin‐70‐14) including a total of 70 lectin proteins, an analysis on glycan expression profiles was conducted using murine *rb1* ^Δ/Δ^ *p53* ^Δ/Δ^ NEPC cells, based on the manufacturer's guidelines. In brief, the cell membrane proteins of *rb1* ^Δ/Δ^ *p53* ^Δ/Δ^ NEPC cells were isolated and followed by incubation with biotin to label the glycoconjugates. The lectin microarrays were then pre‐incubated with a blocking buffer at room temperature for 30 min to mitigate non‐specific binding. After washing and drying procedures, 100 µL diluted sample of cell membrane proteins (at the ratio of 1:30) was applied onto the microarray, with subsequent overnight incubation at 4 °C to allow for lectin‐glycan interactions. The arrays were subsequently rinsed and washed followed by 1 h incubation with 70 µL of secondary antibody in a dark chamber at room temperature to enhance signal detection. Finally, the microarrays were washed, dehydrated, and scanned employing an Olympus BX‐53 microscope to visualize the lectin‐glycan interactions. Other methods and materials are available in Supporting Information.

### Platelet Activation Assay

For platelet extraction, blood was collected via eyeball enucleation from C57BL/6 mice. The blood samples were treated with 5% EDTA and centrifuged at 200 g at RT for 12 min to prevent coagulation. After the centrifuge, the upper layer of blood, which was platelet‐enriched plasma, was transferred into new Eppendorf tubes and centrifuged at 1200 g for 5 min. The supernatant plasma was discarded and the pellets were washed with PBS for 3 times. Next, 1×10^6^ *rb1* ^Δ/Δ^ *p53* ^Δ/Δ^ NEPC or *myc* ^hi^ *pten* ^Δ/Δ^ PrAD tumor cells were incubated with 10 µg mL^−1^ of Mannose‐Binding Protein‐Associated Serine Protease (MBP‐MASP) on ice for 30 min. Tumor cells were then washed with PBS. After that, 50 µL of C3 (200 SFU mL^−1^) complement was added to each tube and incubated with tumor cells for 30 min and followed by supernatant collection. The supernatant was added to the platelets for 15 min incubation on ice. Then, the incubated platelets were stained with flow cytometric antibodies anti‐CD41‐FITC and anti‐CD62P‐PE for 15 min. Finally, PBS was added to terminate the reaction, and platelet activation was analyzed by flow cytometric analysis on CD41 and CD62P double positive (CD41^+^CD62P^+^) cell percentage, and data were processed and presented using the FlowJo software.

### The Tail Vein Bleeding Assay

The tails of mice were swabbed using 75% ethanol to avoid infections. Then, the tails of mice were immersed in 50 °C water for 5 min to ensure that the tail vessels were engorged with blood. Next, the tail tips were dried and trimmed off using a sterilized scissor, resulting in bleeding. Then, count the time from the mice began to bleed until full coagulation.

### RNA Isolation and RT‐qPCR

For RNA isolation, total RNA was purified by the FastPure Cell/Tissue Total RNA Isolation Kit (RC112‐01, Vazyme). Reverse transcription (RT) of RNA to cDNA was conducted using the Hi‐Script II Q RT SuperMix (R223‐1, Vazyme). The RT‐qPCR reaction was performed using SYBR‐green (Q711‐2, Vazyme) reagent based on the manufacturer's instructions. In this study, β‐Actin (*Actb*) was used as the internal control. Data were calculated by ^ΔΔ^Ct which were conducted in biological or technical triplicates. Primers used in RT‐qPCR experiments are listed in Table S1 (Supporting Information).

### Immunoblotting Assay

Cells were lysed using the RIPA lysis buffer (Thermo Fisher) supplemented with Protease Inhibitor Cocktail (PIC, MCE, HY‐K0011). Proteins of interest were separated by SDS‐PAGE and electrophoresis, and transferred onto the polyvinylidene difluoride (PVDF) membranes. PVDF membranes were then blocked with 10% skimmed milk at RT for 1 h and incubated with indicative primary antibodies at 4 °C overnight and followed by horseradish peroxidase (HRP)‐conjugated secondary antibodies for 1 h at RT. Protein bands were detected and visualized using an ECL chemiluminescent HRP substrate detection kit (Millipore, P90720). Primary antibodies used in immunoblotting assay are available in Table S2 (Supporting Information).

### H&E, IHC, IF, and Fibrin Staining Assays

In brief, murine primary prostate tumors or livers with metastatic lesions were fixed with 10% formalin and underwent paraffin‐embedding. H&E, IHC, and IF staining experiments were conducted with 5 µm‐thicken tissue sections. For H&E staining, IHC, and IF staining assays, tumor or liver sections were processed as it was previously described.^[^ ^15^ ^]^

For the Fibrin Staining assay, a commercially available kit (G2040, Solarbio) was used following the manufacturer's protocol. In general, tissue sections were conventionally de‐paraffined, incubated in Hypo solution for 5 min at RT, and washed by distilled water (dH_2_O). Then, the slides were stained with the celestite blue solution for 5 min at RT and washed with dH_2_O. Next, the sections were incubated with Mayer Hematoxylin solution for 5 min at RT, followed by acid differentiation, and rinsed by dH_2_O for 10 min. After that, the slides were slightly washed with 95% ethanol solution. Then, sections were stained with Mathew Yellow solution for 3 min and washed with dH_2_O slightly. Subsequently, the slides were incubated with acid red solution for 10 min at RT and followed by dH_2_O wash. Next, the sections were stained with phosphotungstic acid solution, and the slides under a phase contrast microscopy until the red color in collagen disappeared were checked, and then the slides using dH_2_O were washed. Subsequently, slides were stained with aniline blue solution for 5 min and were washed by a weak acid solution. Next, the sections were dehydrated in 95% ethanol solution for 10 s and next incubated in absolute ethanol for 10 s for 3 times. Slides were transferred into xylene for 5 min for twice and sealed with neutral balsam, and followed by microscopic inspection and image acquisition. For the IF staining assay, images were captured using a Leica SP8 confocal fluorescence microscope. For H&E, IHC, and Fibrin staining assays, images were obtained using an Olympus BX‐53 microscope. The primary antibodies used in IHC and IF were summarized in Table S2 (Supporting Information).

All human PCa clinical samples were obtained from the Department of Urology of Renji Hospital abide by the national ethical regulations and were approved by the Renji Hospital Ethics Committee. All experimental procedures (KY2024‐076‐B) were approved by the Ethics Committee of Ren Ji Hospital, Shanghai Jiao Tong University School of Medicine.

### Flow Cytometry and Cell Sorting

Dissociated single cells from murine prostate tumors were chopped into small fragments for enzymatic digestion (Dispase, 0.02 mg mL^−1^; DNase I, 0.01 mg mL^−1^; Collagenase I and IV, 0.2 mg mL^−1^; 2% FBS in RIPA‐1640 medium) at 37 °C for 3 h, followed by additional digestion in 0.25% Trypsin‐EDTA on ice for 30 min. Next, cell suspensions were passed through a 70 µm cell strainer. Cells were incubated with anti‐CD16/32 antibody for 30 min on ice to block Fc receptors. Cells were then incubated with indicative florescence conjugated flow antibodies at 4 °C for 30 min. Flow cytometric analyses were performed on BD LSR Fortessa instrument. Flow cytometric data were processed and visualized by the FlowJo software. The flow cytometric antibodies were listed in Table S2 (Supporting Information).

### Co‐Immunoprecipitation (Co‐IP)

The Co‐IP experiments were conducted as it was previously reported.^[^ ^33^ ^]^ In general, cells were lysed by Co‐IP lysis buffer (150 mm NaCl, 50 mm Tris‐HCl, pH 7.5, 1% NP‐40, 5 mm EDTA, and Protease Inhibitor Cocktail). Cell lysates were incubated with indicative antibodies (1: 100 diluted), or anti‐IgG antibodies as control, for immunoprecipitations at 4 °C overnight and followed by incubation with magnetic protein A/G agarose beads (Selleck, B23202) for 2 h at 4 °C. Finally, the immunoprecipitated samples were collected and washed three times using Co‐IP lysis buffer and then detected and analyzed by immunoblotting assays. The antibodies used in Co‐IP assays were listed in Table S2 (Supporting Information).

### Plasmid Construction

The shANXA2 plasmids (5’ CGAGACAAGGTCCTGATTAGA 3’) were constructed using the pLV‐eGFP‐puromycin vector. A full‐length ANXA2 plasmid was also designed and constructed using a V6‐CMV‐HA‐tagged vector (GenScript, Nanjing, China) with the corresponding synonym mutation, which was refractory to shANXA2‐mediated knockdown. Based on this full‐length ANXA2 plasmid, point mutations including serine (S) mutation to alanine (A) at positions 18 (S18A), 22 (S22A), and 26 (S26A) were further introduced.

### RNA‐seq and Bioinformatics

For bulk RNA‐seq, the RNA from primary tumors or/and liver metastatic foci were isolated using the Qiagen RNeasy extraction Kit according to manufacturer's instructions. Subsequently, the RNA‐seq libraries were established using the NEB‐Next UltraTM RNA Library Prep Kit applied for Illumina platforms. The adapter sequences were removed with the Cutadapt software (version 2.6), ensuring that only the purified reads were aligned to the reference genome, version GRCm38 release 84, using Hisat2 aligner with its standard parameters. The measurement of gene expression levels was conducted using the Stringtie tool (version 1.3.6). The identification of genes with significant differences in expression was conducted through the DESeq2 package (version 1.24.0). To subclassify PrAD and NEPC in published SU2C and Beltran prostate cancer datasets, the AR score‐related genes were referred to a gene set from the cBioPortal database, encompassing a total of 30 AR‐responsive genes. The 70 NE‐related genes also referred to a gene set from the cBioPortal database (www.cbioportal.org). Patient‐derived RNA‐seq data from SU2C (accession number: phs000915.v2.p2) and Beltran's (accession number: phs000909.v1.p1.c1) datasets for prostate cancer were retrieved from the database of Genotypes and Phenotypes (dbGaP). For Gene Set Enrichment Analysis (GSEA) was conducted using the GSEA (version 4.0.3) software in combined with the Molecular Signatures Database (MSigDB, version 7.1). For KEGG and GO analyses, the limma R package (version 3.56.2) was utilized to screen differentially expressed genes (DEGs), and identified gene sets that significantly changed under different experimental conditions. Subsequently, the cluster profile R package (version 4.8.3) was employed to compare and enrich indicative gene sets in KEGG and GO databases.

### Statistics Analysis

All statistical analyses in this study were conducted using the GraphPad 8.0 software. In brief, the patient survival was analyzed by the Kaplan–Meier analysis and curves of MBL‐low and MBL‐high groups were compared using a log‐rank test. For RT‐qPCR results and other quantification results, the student's *t‐*test was used for two‐group experimental data comparison. For multiple‐group data, the one‐way ANOVA, or two‐way ANOVA test was used to assess significant differences. Data were represented means ± SD. For all statistical tests, *p*‐value < 0.05 was regarded as statistically significant.

## Conflict of Interest

The authors declare no conflict of interest.

## Author Contributions

X.C. and W.B. contributed equally to this work. X.C and W.B. performed most of the experiments and contributed equally to this work. K.L., N.J., G.D., L.J., Q.Y., and Y.Z. assisted in organoid culture and cell line establishment. P.X. and C.C. assisted in creating cartoon elements in Graphical abstract drawing. Y.L. assisted in in vivo experiments and sample collections. J.W. provided instructions in bioinformatic analyses. H.Z. assisted in flow cytometric analyses. S.Z, D.W, C‐F.N, J.P, W.X, W.Q.G, and P.Z provided kind suggestions and important discussion during manuscript preparation. K.Z conducted most IHC staining assays. H.H.Z, K.Z, and X.C interpreted the data. H.H.Z., K.Z., and X.C. wrote the manuscript. H.H.Z. and K.Z. were funded and supervised in this study. H.H.Z conceived this study.

## Supporting information



Supporting Information

## Data Availability

The data that support the findings of this study are available from the corresponding author upon reasonable request.
