# Supplementary material for: O‐GalNAc Glycosylation Activates MBL‐Mediated Complement and Coagulation Cascades to Drive Organotropic Metastasis
Source: Adv Sci (Weinh). 2025 Jun 10;12(32):e04809. doi: 10.1002/advs.202504809 (PMC12407318; doi:10.1002/advs.202504809)
Supplement: Supplementary file 1 — Supporting Information [file ADVS-12-e04809-s001.docx]

**Supporting Information for**

**O-GalNAc** **Glycosylation Activates MBL-Mediated** **Complement and Coagulation Cascades to Drive Organotropic Metastasis**

**Authors:** Xinyu Chen^1#^, Wei Bao^1#^, Kaiyuan Liu^1^, Na Jing^1^, Genyu Du^1^, Luyao Jiang^1^, Qian You^1^, Yingchao Zhang^1^, Penghui Xu^1,2^, Chaping Cheng^1^, Nan Wang^1^, Xialian Xi^1^, Mingyue Wang^1^, Yiyun Liu^1^, Jinming Wang^1^, Huifang Zhao^1^, Shilei Zhang^3^, Dinglan Wu^4^, Chi-Fai Ng^5^, Jiahua Pan^6^, Wei Xue^6^, Wei-Qiang Gao^1,2^, Pengcheng Zhang^7^, Kai Zhang^1*^, and Helen He Zhu^1*^

**Affiliations:**

^1^State Key Laboratory of Systems Medicine for Cancer, Department of Urology, Ren Ji Hospital, Shanghai Cancer Institute, Shanghai Jiao Tong University School of Medicine, Shanghai 200127, China;

^2^Med-X research Institute, School of Biomedical Engineering, Shanghai Jiao Tong University, Shanghai 200030, China.

^3^Department of Pathology, Ren Ji Hospital, Shanghai Jiao Tong University School of Medicine, Shanghai 200127, China

^4^Department of Surgery, Faculty of Medicine, The Chinese University of Hong Kong, Hong Kong, China

^5^S.H. Ho Urology Centre, Department of Surgery, Prince of Wales Hospital, The Chinese University of Hong Kong, Hong Kong, China.

^6^Department of Urology, Renji Hospital, Shanghai Jiao Tong University School of Medicine, Shanghai 200127, China;

^7^School of Biomedical Engineering, Shanghai Tech University, Shanghai, China

^#^ These two authors contribute equally to this paper.

**Correspondence***: Helen He Zhu ([zhuhecrane@shsmu.edu.cn](mailto:zhuhecrane@shsmu.edu.cn)), and Kai Zhang ([zhangkaishida@126.com](mailto:zhangkaishida@126.comalumni.sjtu.edu.cn)), Tel: 86-21-62932049, Fax: 86-21-68383916.

**Supplementary Tables**

**Table S1: Primers used in RT-qPCR**

| Primers | Species | Sequence (5’ to 3’) |
| --- | --- | --- |
| GALNT9-F | human | GGAAGCCCTACAACAACGACA |
| GALNT9-R | human | GGGTTCGACATGGGGATGTTC |
| Galnt9-F | mouse | GACCGAGCCATCCTCTACC |
| Galnt9-R | mouse | CACTTGGAGTCGGGCAGAAA |
| ANXA2-F | human | TCTACTGTTCACGAAATCCTGTG |
| ANXA2-R | human | AGTATAGGCTTTGACAGACCCAT |
| Anxa2-F | mouse | ATGTCTACTGTCCACGAAATCCT |
| Anxa2-R | mouse | CGAAGTTGGTGTAGGGTTTGACT |
| ACTB-F | human | CACCATTGGCAATGAGCGGTTC |
| ACTB-R | human | AGGTCTTTGCGGATGTCCACGT |
| Actb-F | mouse | GGCTGTATTCCCCTCCATCG |
| Actb-R | mouse | CCAGTTGGTAACAATGCCATGT |

**Table S2: Antibodies used in this study**

| Protein | Brand | Catalog No: | Clone | Dilution |
| --- | --- | --- | --- | --- |
| CD41-FITC | Invitrogen | 11-0411-82 | eBioMW/Reg30 | 1:100 for Flow |
| CD62p-PE | Invitrogen | 12-0626-82 | Psel.KO2.3 | 1:100 for Flow |
| ANXA2 | santa cruz | sc-28385 | C-10 | 1:1000 for WB |
| GALNT9 | NOVUS | NBP2-16598 | Polyclonal | 1:1000 for WB; 1:500 for IHC |
| O-GalNAC | sigma-aldrich | SAB5202328 | 9B9 | 1:1000 for WB; 1:500 for IHC/IF |
| CD41 | proteintech | 24552-1-AP | Polyclonal | 1:500 for IHC |
| Fibrin | Sigma-Aldrich | MABS2155 | 59D8 | 1:500 for IHC |
| HA-tag | ABclonal | AE008 | Polyclonal | 1:1000 for WB |
| ACTIN | ABclonal | AC026 | Polyclonal | 1:1000 for WB |
| CD42b | Emfret | R300 | polyclonal | 2 µg/g in vivo |
| Ki67 | Abcam | Ab15580 | polyclonal | 1:500 for IHC |
| Synaptophysin | Abcam | ab32127 | YE269 | 1:500 for IHC |
| CD56 | Cell Signaling Technology | 99746S | E7X9M | 1:500 for IHC |

**Table S3: Primers used in genotyping (from 5’ to 3’)**

| *Pbsn-cre-F* | CTGAAGAATGGGACAGGCATTG |
| --- | --- |
| *Pbsn-cre-R* | CATCACTCGTTGCATCGACC |
| *Pten-F* | CAAGCACTCTGCGAACTGAG |
| *Pten-R* | AAGTTTTTGAAGGCAAGATGC |
| *Trp53-F* | GGTTAAACCCAGCTTGACCA |
| *Trp53-R* | GGAGGCAGAGACAGTTGGAG |
| *Rb1-F* | CTCATGGACTAGGTTAAGTTGTGG |
| *Rb1-R* | GCATTTAATTGTCCCCTAATCC |
| *Hi-Myc-F* | GCATTGGGCATTGTCCATGCCTA |
| *Hi-Myc-R* | AGAAGGGTGTGACCGCAACGTA |
| *JS09442-Mbl2-5wt-tF1* | GGGCTGAAGTTACAGGCAGTTG |
| *JS09442-Mbl2-3wt-tR1* | CTTTGCCCACCACCACCAA |
| *JS19442-Mbl2-wt-tF1* | GTCTGACTCCAGGATGCCAGTTC |
| *JS19442-Mbl2-wt-tR1* | CTTGAGACCCATTGTGAGATGCTT |

**Table S4: shRNA sequences used in this study**

| sh*Galnt9*-1# | GTGTACATGGCATGGAATA |
| --- | --- |
| sh*Galnt9*-2# | ACGTACGGAGAGGTGAGAA |
| sh*GALNT9*-1# | ACAACGACATTGACTACTA |
| sh*GALNT9*-2# | AACAACGACATTGACTACT |
| sh*Anxa2*-1# | CGAGACAAGGTCCTGATTAGA |
| sh*Anxa2*-2# | AGTTATTGACTACGAGCTGAT |

**Table S5: The Clinical information of PCa patient samples**

| **Patient ID** | **GALNT9 intensity (IOD/area)** | **O-GalNAc intensity (IOD/area)** | **Histology** |
| --- | --- | --- | --- |
| 1# | 184 | \| 101 \| \| --- \| | NEPC |
| 2# | 97 | \| 109 \| \| --- \| | NEPC |
| 3# | 136 | 123 | NEPC |
| 4# | 27 | 12 | PrAD |
| 5# | 34 | 30 | PrAD |
| 6# | 36 | 35 | PrAD |

The clinical information of the tumor sections of our in-house PCa patients in the current study. The IHC intensity of GALNT9 was calculated using Image J software by measuring IOD/area. (**PrAD**: prostate adenocarcinoma; **NEPC**：neuroendocrine prostate cancer; **IOD**: integral optical density)

**Supplementary Figures**

**Figure S1: Coagulation related pathways are significantly activated in NEPC liver metastasis versus primary tumors in mice and humans.**


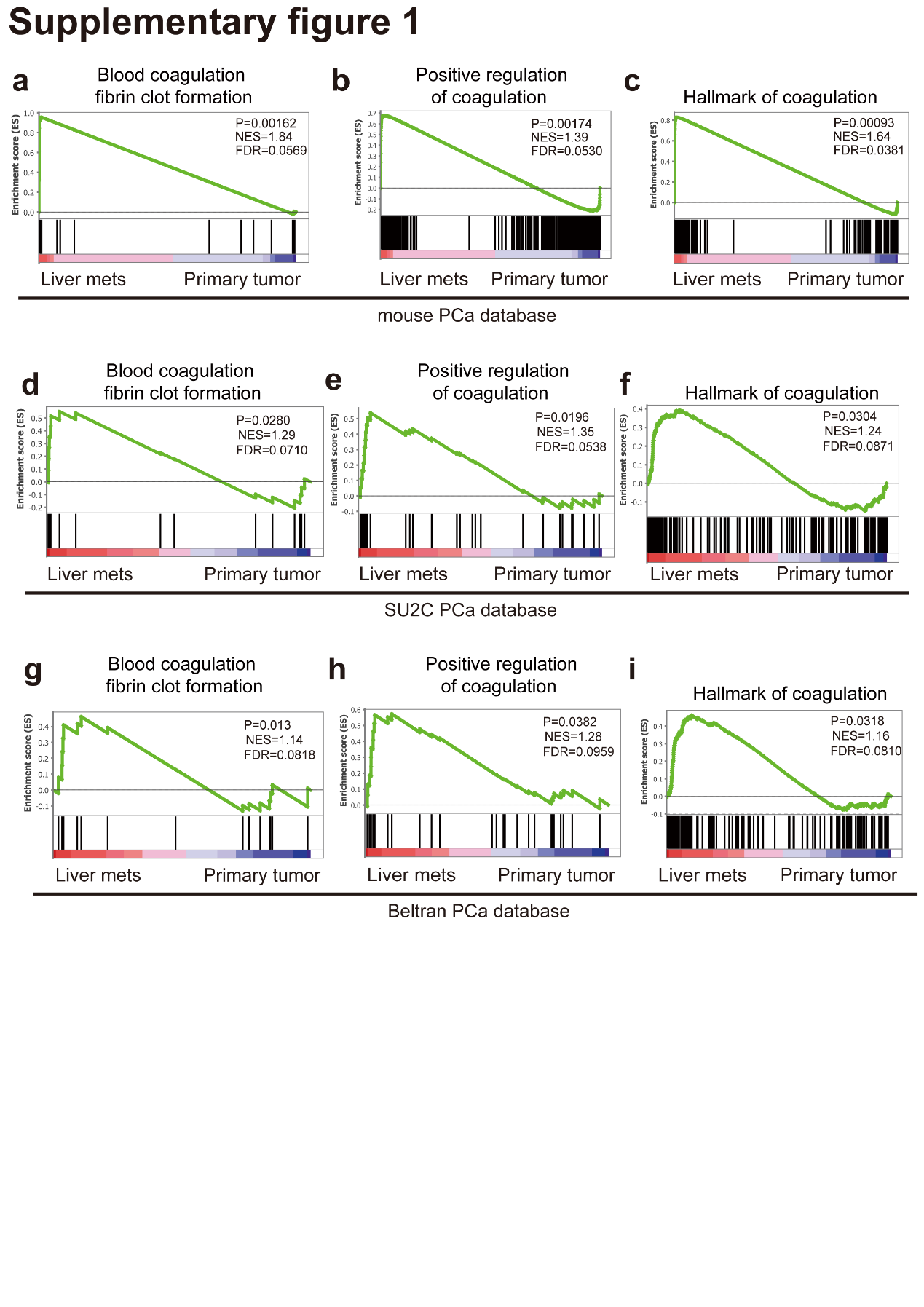


**(a-c)** GSEA plots showing that “Blood coagulation fibrin clot formation” **(a)**, “Positive regulation of coagulation” **(b)** and “Hallmark of coagulation” signaling pathway **(c)** were all significantly upregulated in liver metastasis comparing to primary tumors of *rb1*^Δ/Δ^*p53*^Δ/Δ^ NEPC-tumor bearing mice via our RNA-seq dataset.

**(d-f)** GSEA plots suggesting that “Blood coagulation fibrin clot formation” **(d)**, “Positive regulation of coagulation” **(e)** and “Hallmark of coagulation” signaling pathway **(f)** were all significantly upregulated in liver metastasis comparing to prostate primary tumors based on human SU2C PCa database.

**(g-i)** GSEA plots revealing that “Blood coagulation fibrin clot formation” **(g)**, “Positive regulation of coagulation” **(h)** and “Hallmark of coagulation” signaling pathway **(i)** were all significantly upregulated in liver metastasis comparing to prostate primary tumors based on human Beltran PCa database.

**Figure S2: The MBL pathway, rather than the classical or alternative pathway, is significantly activated in liver metastasis of NEPC.**

**
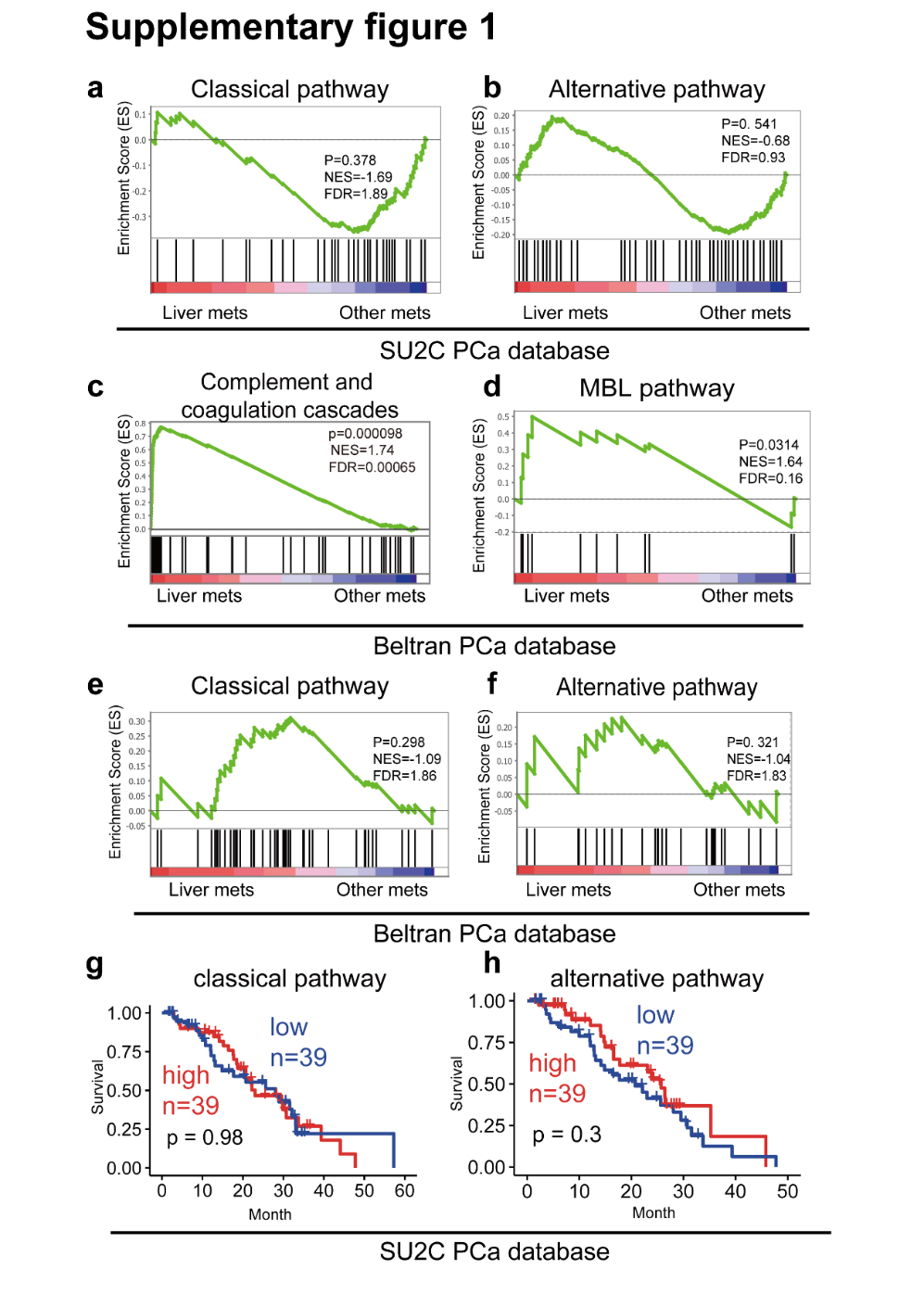
**

**(a-b)** GSEA plots showing that neither classical **(a)** nor alterative pathway **(b)** was significantly upregulated in liver metastasis comparing to other metastatic lesions based on SU2C human prostate cancer dataset.

**(c-d)** GSEA plots revealing that the “complement and coagulation cascades” **(c)** and “MBL pathway” **(d)** were significantly upregulated in liver metastasis in comparison to other metastatic lesions based on Beltran human PCa dataset.

**(e-f)** GSEA plots showing that neither classical **(e)** nor alterative pathway **(f)** was significantly upregulated in liver metastasis comparing to other metastatic lesions based on Beltran human PCa dataset.

**(g-h)** Based on human SU2C PCa dataset, neither the classical **(g)** nor alterative pathway **(h)** activity was significantly correlated with patients’ survival.

**Figure S3: O-GalNAc is the most upregulated glycosylation in NEPC compared to PrAD patients.**

**
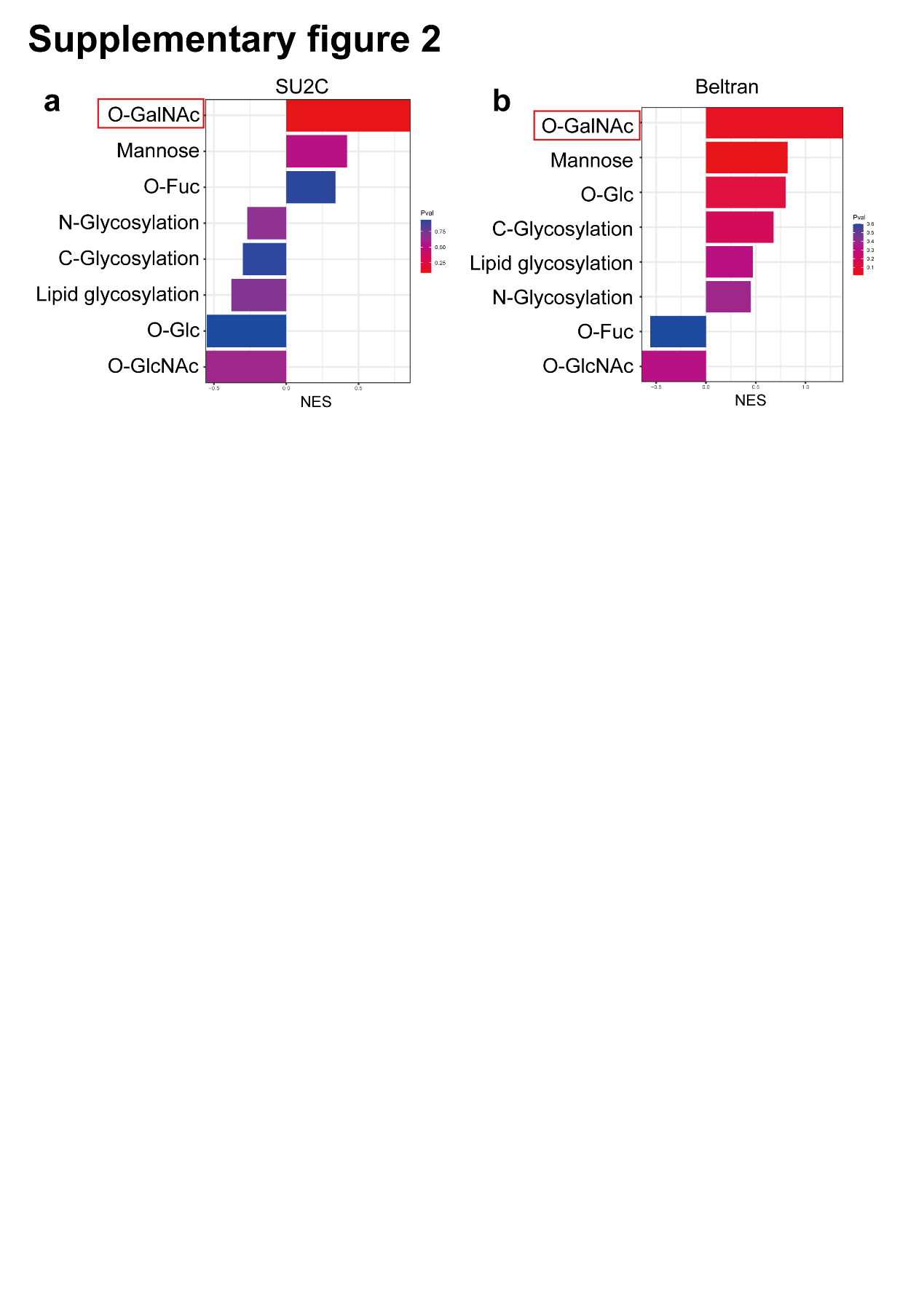
**

**(a-b)** KEGG analyses showing the most significantly upregulated glycosylation forms in NEPC versus PrAD based on human SU2C (**a**) and Beltran (**b**) PCa databases.

**Figure S4: Galnt9 expression and O-GalNAc glycosylation levels are higher in NEPC compared to PrAD in clinical specimens.**


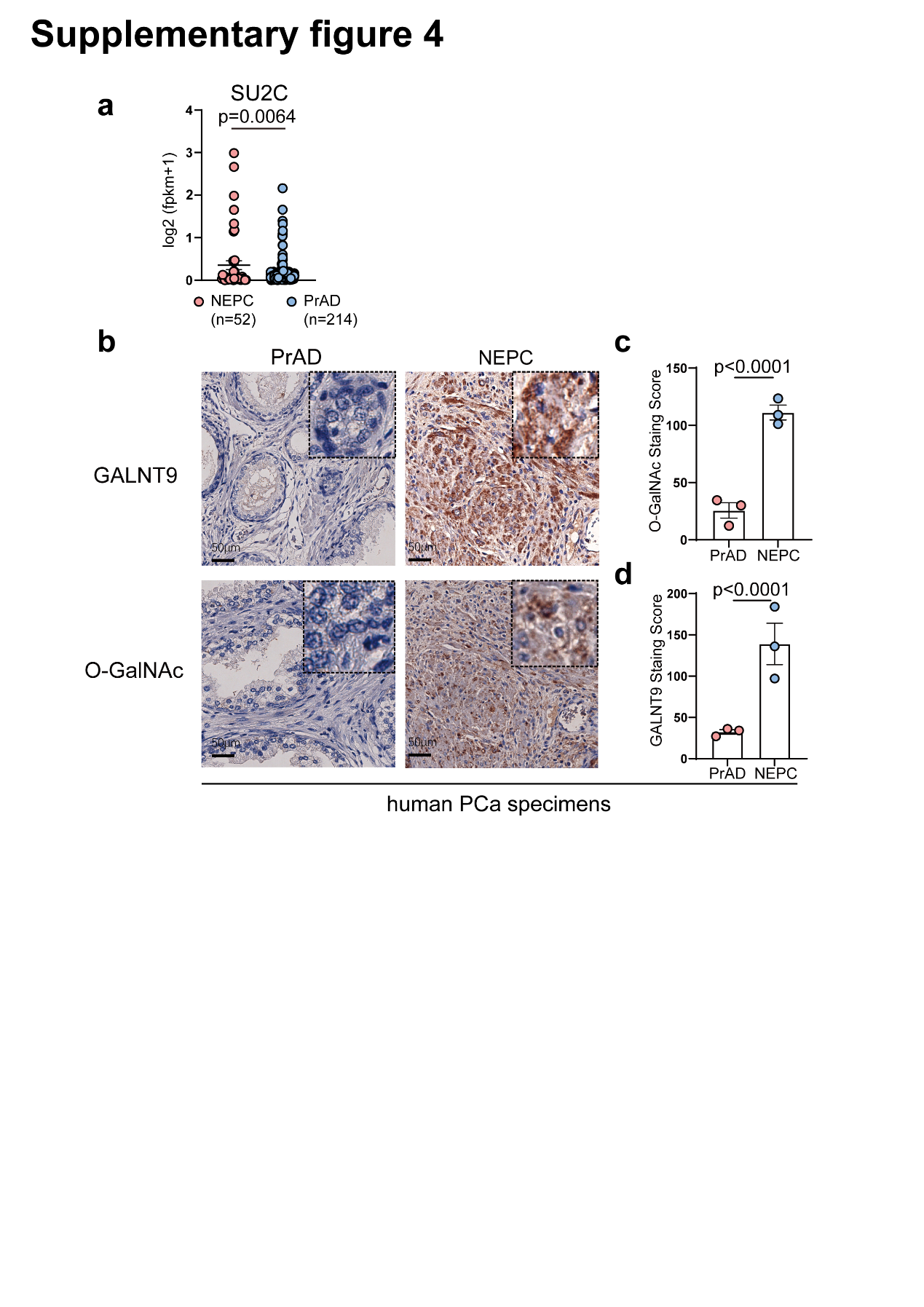


**(a)** Based on human SU2C PCa dataset, NEPC patients (n = 52) expressed significantly higher level of *GALNT9* than PrAD counterparts (n = 214).

**(b-d)** IHC staining **(b)** against GALNT9 (upper panel) and O-GalNAc (lower panel) and quantification results **(c-d)** showing that clinical NEPC biospecimens expressed higher levels of GALNT9 **(b-c)** and O-GalNAc **(b** and **d)** than PrAD patient samples. Scale bar in **(e)** = 50 μm.

For statistics in this figure, the two-tail unpaired Student’s-*t* test was applied for **(a)** and **(c-d)**. Data were shown as means ± SD.

**Figure S5: Galnt9 and O-GalNAc glycosylation are upregulated in SCLC compared to LUAD.**


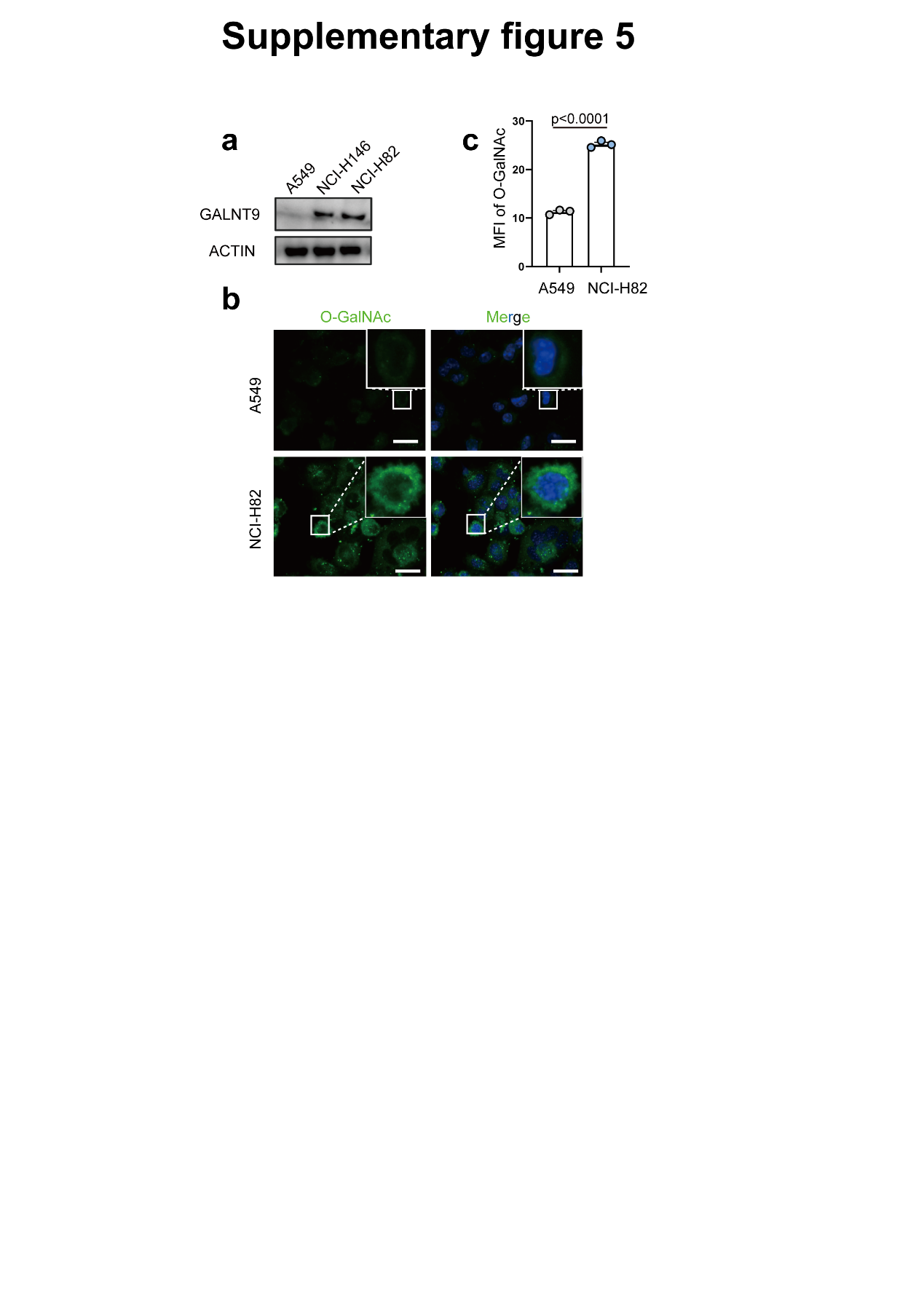


**(a)** Immunoblots showing that GALNT9 is elevated in two human SCLC cell lines including NCI-H146 and NCI-H82 in comparison to human LUAD cell line A549.

**(b-c)** IF staining images **(b)** and MFI quantification results **(c)** revealing that the O-GalNAc glycosylation level was significantly elevated in human SCLC cell line NCI-H82 cells compared to human LUAD cell line A549.

For statistics in this figure, the two-tail unpaired Student’s-*t* test was applied for **(c)**. Data were shown as means ± SD.

**Figure S6: Validation of Galnt9 knockdown (Galnt9-KD) in NEPC, SCLC, and neuroendocrine colon cancer cell lines.**

**
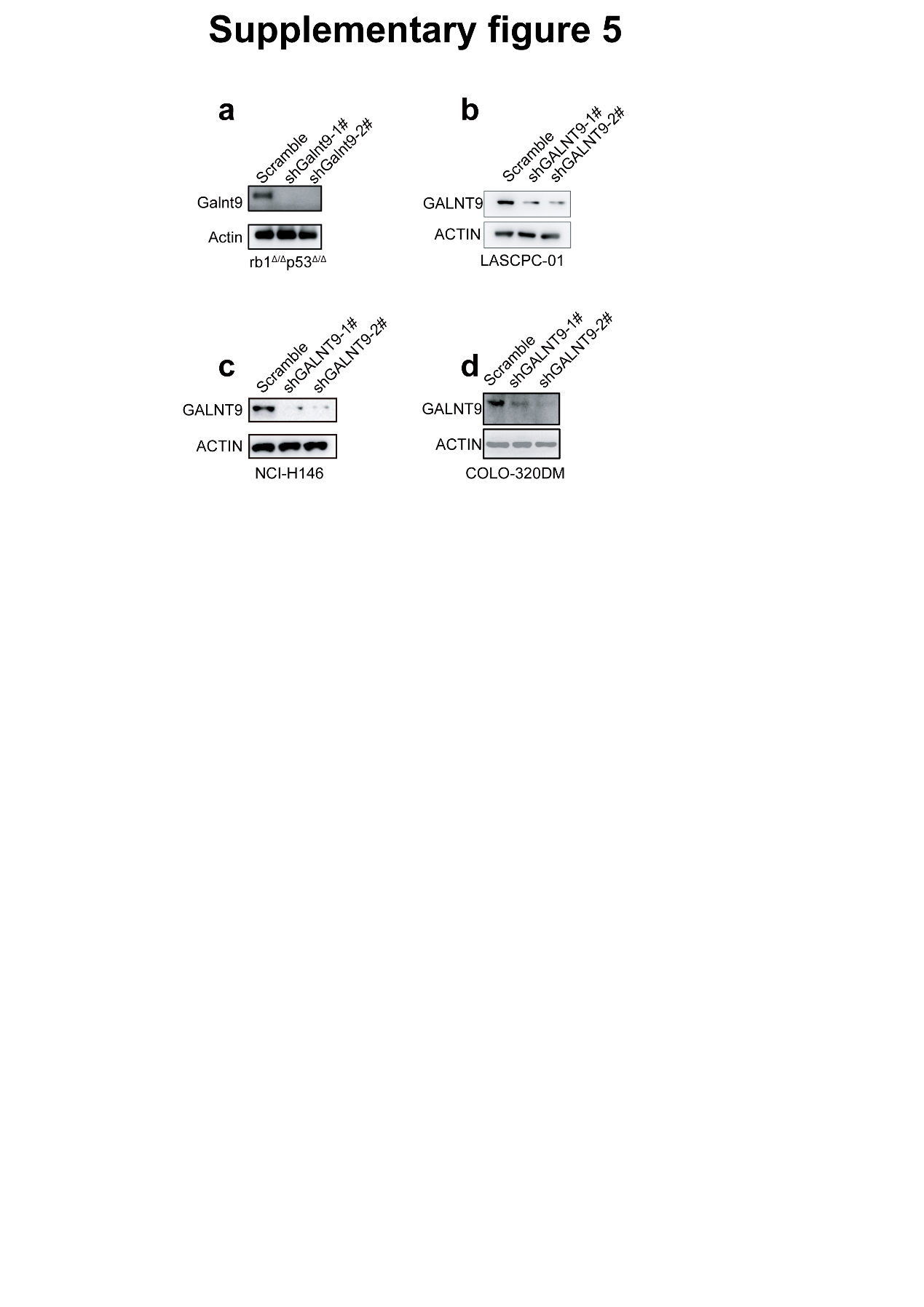
**

**(a-d)** Immunoblots revealing the Galnt9-KD efficiency in murine *rb1*^Δ/Δ^*p53*^Δ/Δ^ NEPC **(a)**, human NEPC LASCPC-01 cells **(b)**, small cell lung cancer NCI-H146 cells **(c)**, and COLO-320DM cells **(d)**, respectively.

**Figure S7: GALNT9 is required for MBL binding and activation and liver metastasis in SCLC and neuroendocrine colon cancers.**


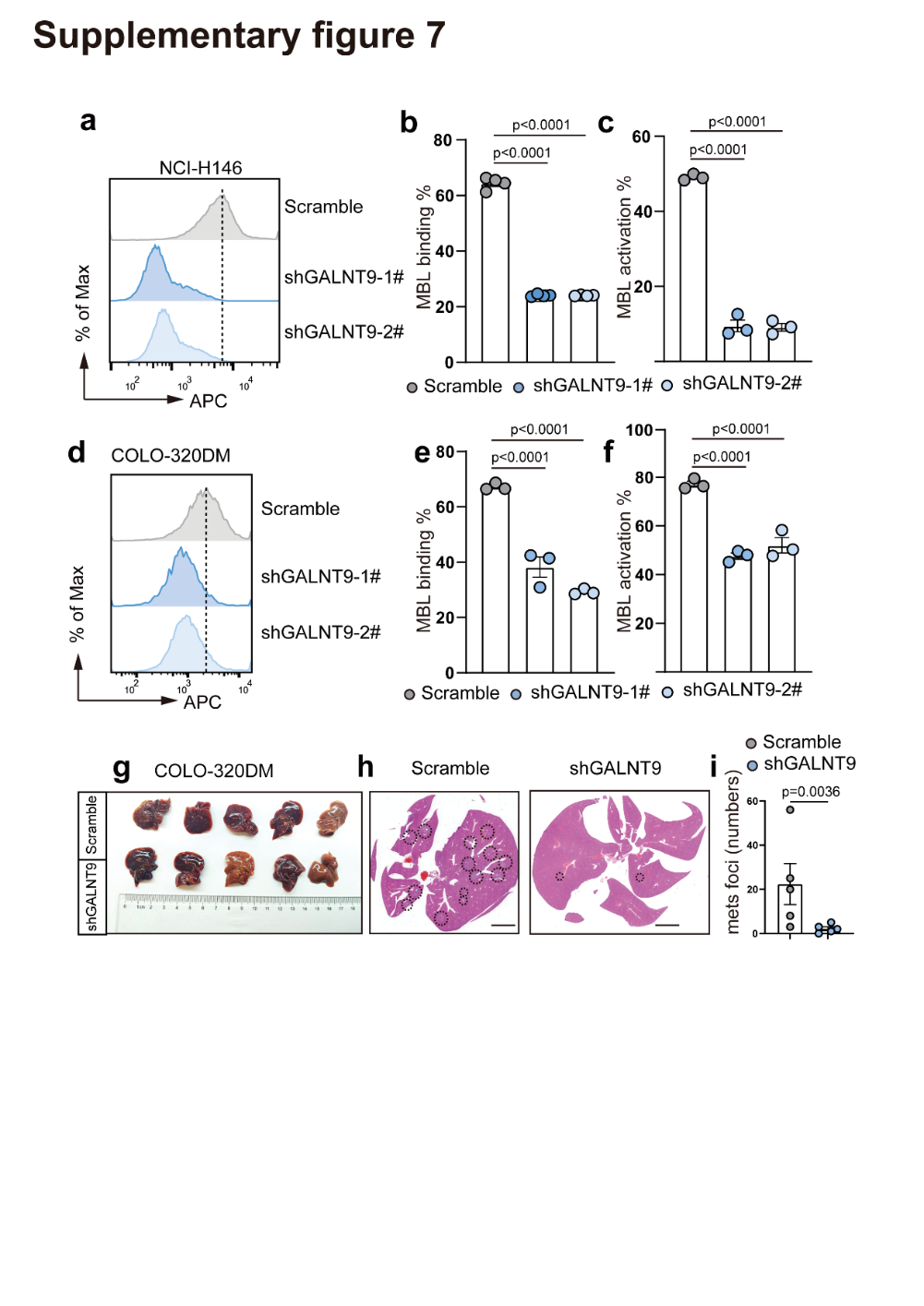


**(a-c)** GALNT9-KD in SCLC NCI-H146 cells led to significantly reduced MBL binding **(a-b)** and activation **(c)** capabilities as compared to scramble control.

**(d-f)** GALNT9-KD in neuroendocrine colon cancer cells COLO-320DM cells resulted in significantly reduced MBL binding **(d-e)** and activation **(f)** capabilities as compared to scramble control.

**(g-i)** In vivo experimental data showing that COLO-320DM-shGALNT9 cells incurred attenuated liver metastatic burdens **(g-h)**, as exemplified by significantly reduced foci number **(i)** in nude mice compared to COLO-320DM-scramble counterparts (n=5, mice).

For statistics in this figure, the two-tail unpaired Student’s-*t* test was applied for **(i),** and the one-way ANOVA test was applied for **(b-c)** and **(e-f)**. Data were shown as means ± SD.

**Figure S8: Ectopic expression of Galnt9 in LUAD leads to elevated MBL binding and activation.**


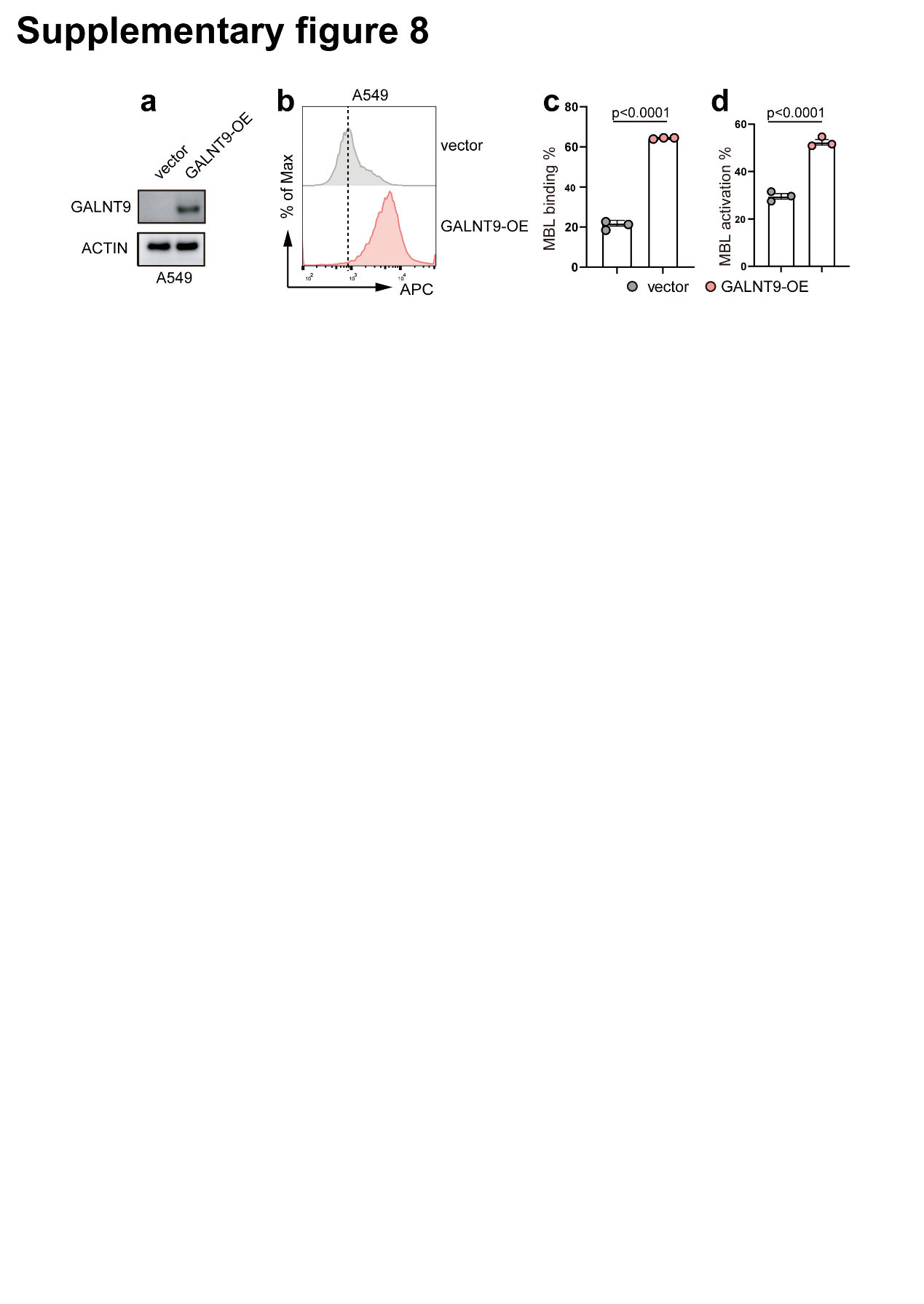


**(a)** Immunoblots confirming that GALNT9 was indeed overexpressed (GALNT9-OE) in human LUAD A549 cells.

**(b-d)** GALNT9-OE in A549 cells results in significantly increased MBL binding **(b-c)** and activation **(d)** capabilities compared to vector controls.

For statistics in this figure, the two-tail unpaired Student’s-*t* test was applied for **(c-d)**. Data were shown as means ± SD.

**Figure S9: Galnt9 is required for platelet activation in NEPC.**


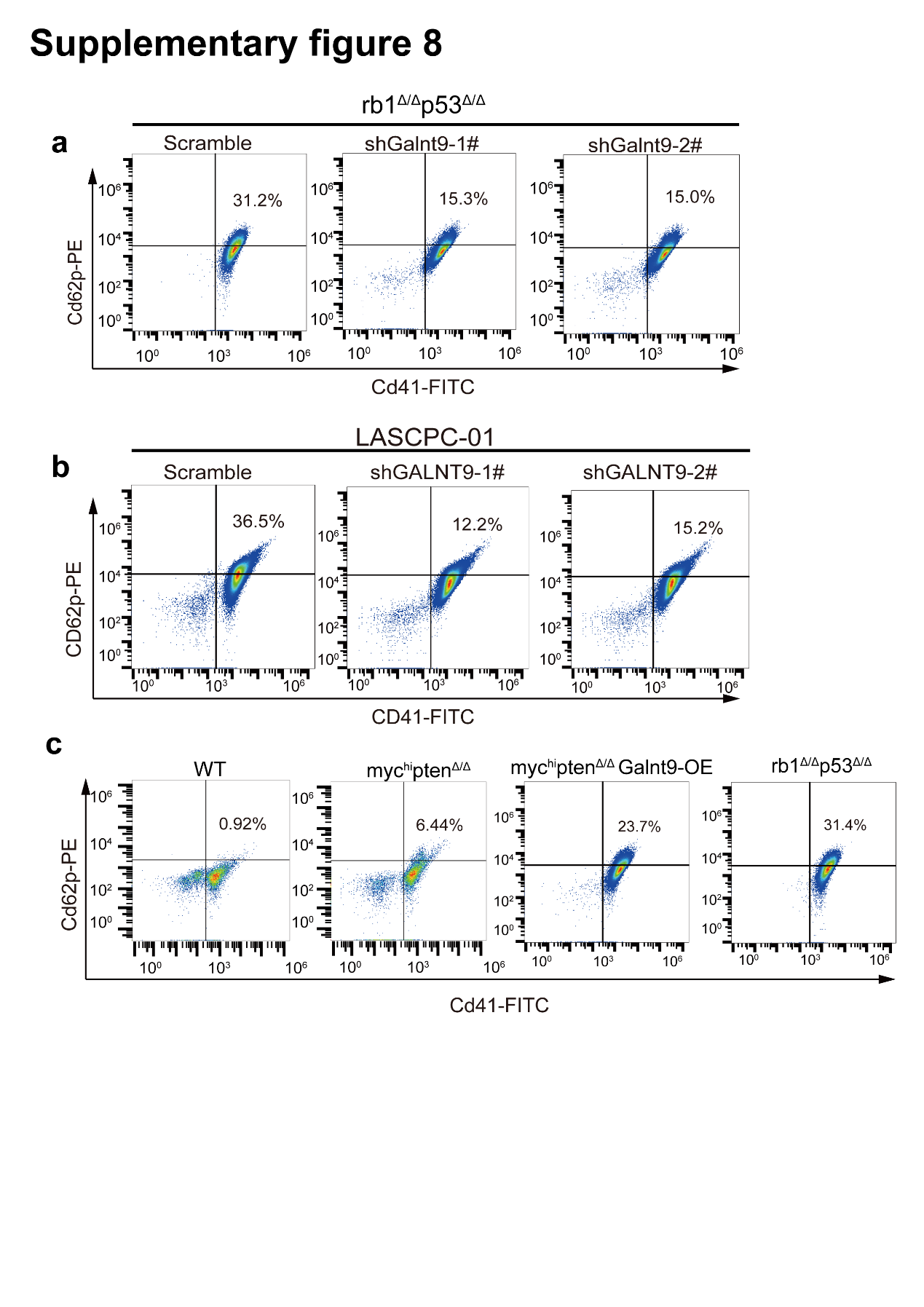


**(a-b)** Representative flow cytometric plots showing that GALNT9-KD in either murine *rb1*^Δ/Δ^*p53*^Δ/Δ^ **(a)** or human LASCPC-01 **(b)** NEPC cells leads to significantly reduced platelet activation capabilities compared to scramble control.

**(c)** Representative flow cytometric plots showing that Galnt9-OE in *myc*^hi^*pten*^Δ/Δ^ PrAD cells significantly enhanced platelet activation capabilities compared to vector control.

**Figure S10: Proteinase K treatment significantly inhibits MBL binding of murine *rb1*^Δ/Δ^*p53*^Δ/Δ^ NEPC organoids.**


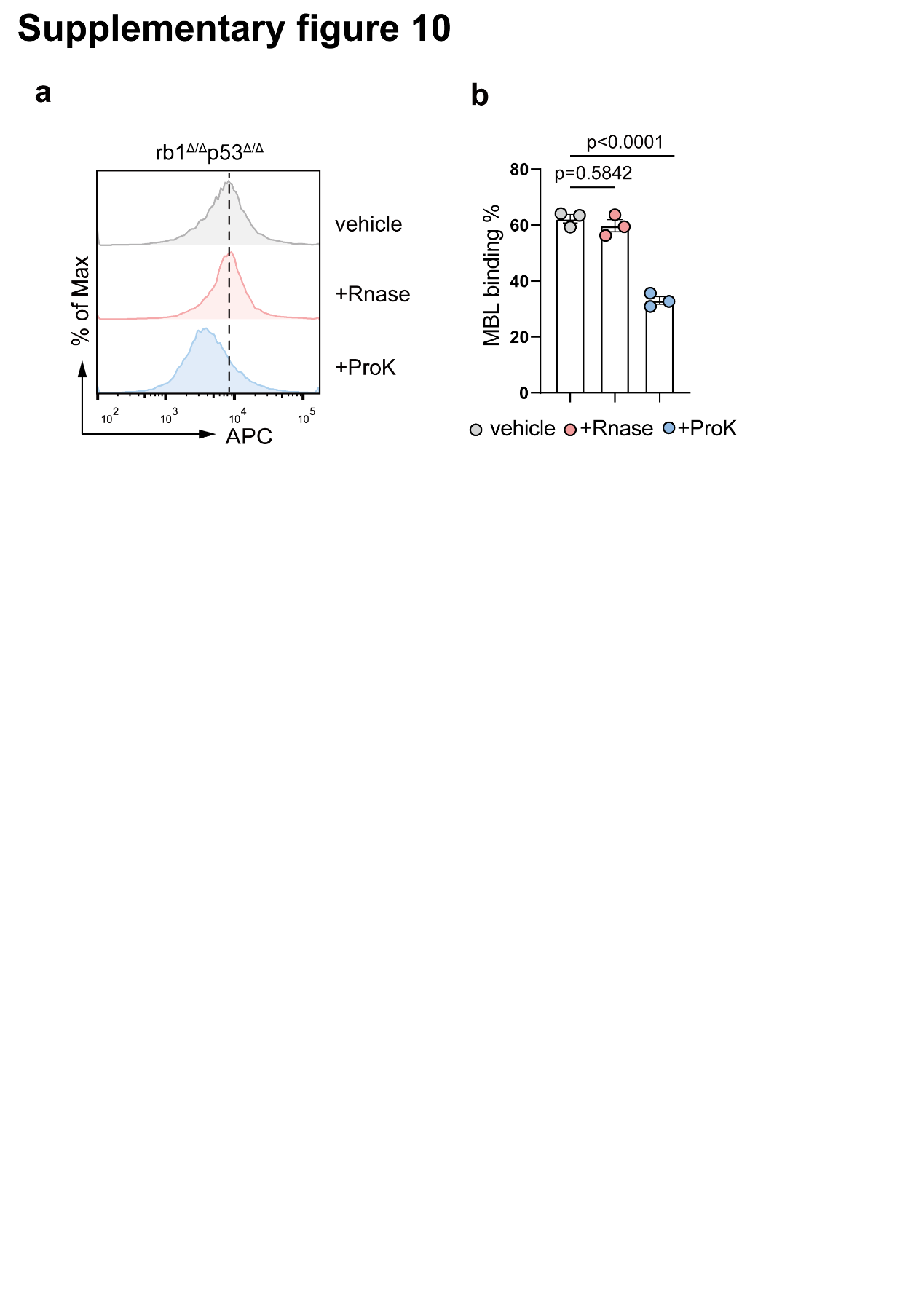


**(a-b)** The flow cytometric plots **(a)** and quantification data **(b)** showing that MBL binding activity of *rb1*^Δ/Δ^*p53*^Δ/Δ^ NEPC organoids was significantly dampened upon proteinase K treatment, but not RNase.

For statistics in this figure, the one-way ANOVA test was applied for **(b)**. Data were shown as means ± SD.

**Figure S11: ANXA2 protein is highly expressed in NEPC versus PrAD in patient samples.**


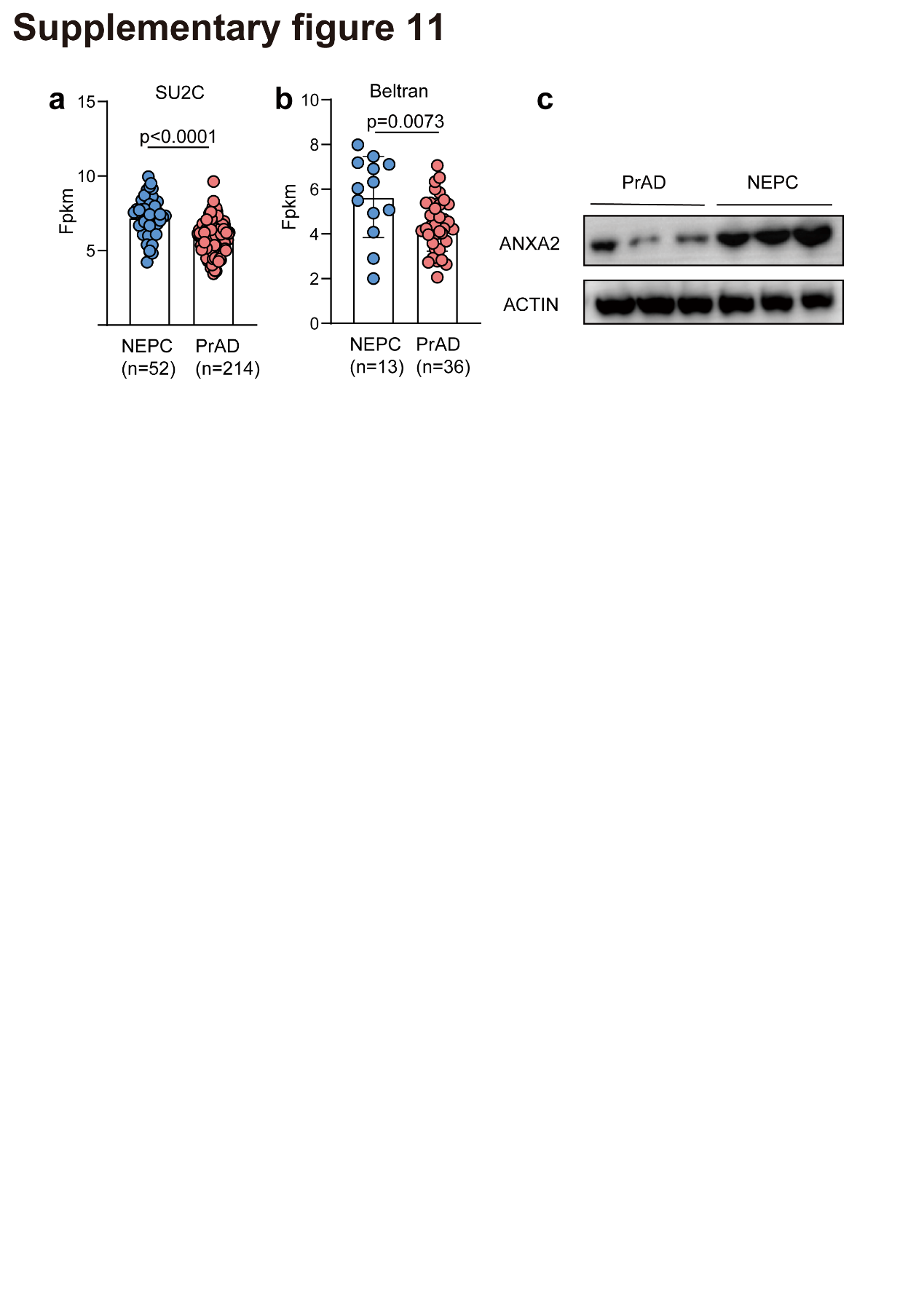


**(a-b)** *ANXA2* mRNA levels in human PrAD and NEPC patients based on SU2C (NEPC, n = 13 patients; PrAD, n = 36 patients) (**a**) and Beltran (NEPC, n = 52 patients; PrAD, n = 214 patients) (**b**) PCa datasets.

**(c)** Immunoblots confirming that ANXA2 was higher in 3 cases of human NEPC samples than those in PrAD tissues.

For statistics in this figure, the two-tail unpaired Student’s-*t* test was applied for **(a-b)**. Data were shown as means ± SD.

**Figure S12: A graphical abstract illustrating that Galnt9-mediated O-GalNAc Glycosylation activates the MBL complement and coagulation cascades to drive liver organotropic metastasis in neuroendocrine carcinomas.**


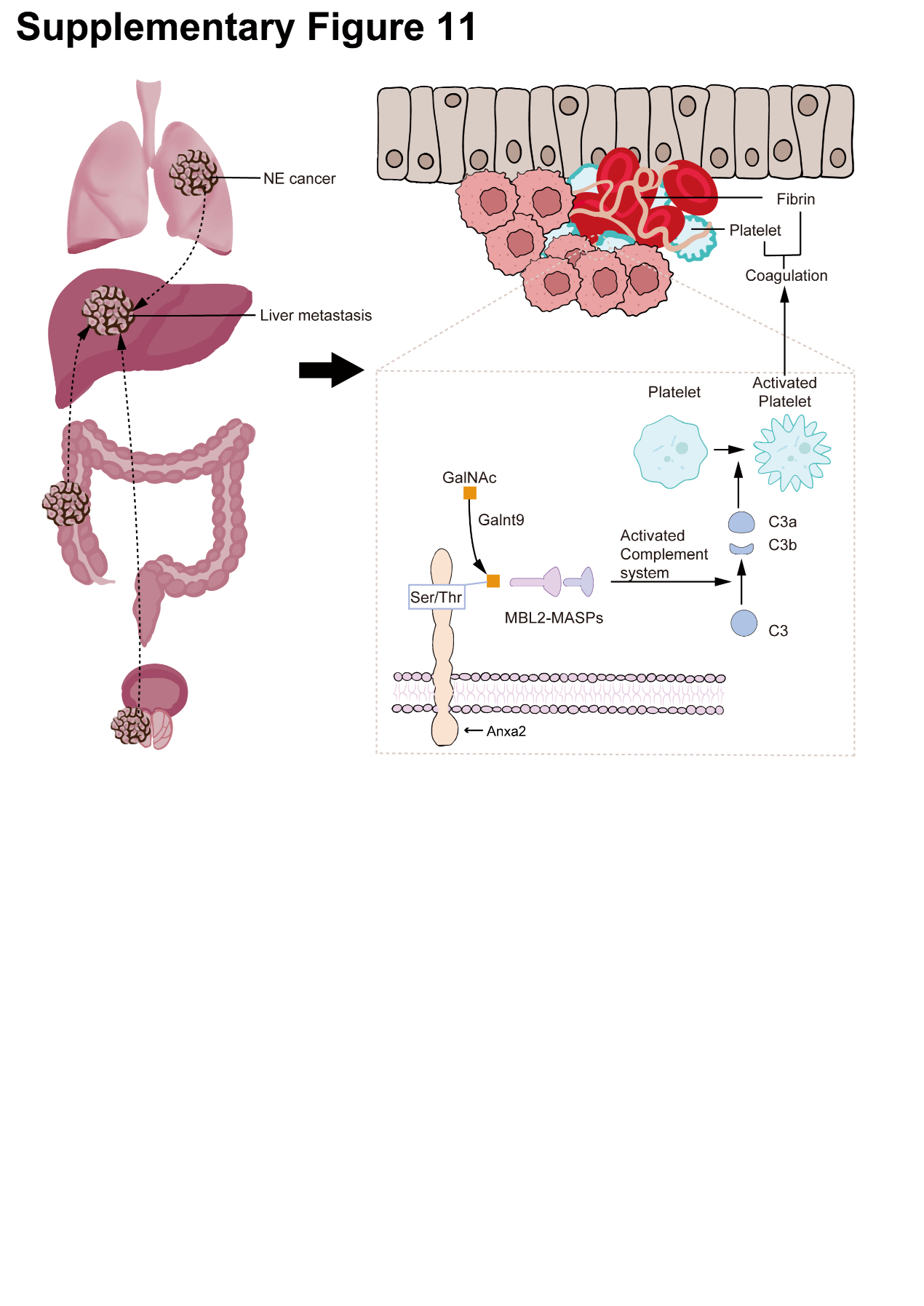


Upregulation of GALNT9 in neuroendocrine carcinomas increases O-GalNAc glycosylation on cell membrane proteins, particularly ANXA2. The aberrantly elevated O-GalNAc glycan induces the binding of neuroendocrine cancer cells to MBL2, activating the MBL-MASP complement pathway in the liver. This cascade subsequently triggers platelet activation and thrombus formation, facilitating liver metastasis in NEPC, SCLC, and neuroendocrine colon cancers.
